# Maturation of the Locomotor Circuitry in Children With Cerebral Palsy

**DOI:** 10.3389/fbioe.2020.00998

**Published:** 2020-08-18

**Authors:** Germana Cappellini, Francesca Sylos-Labini, Arthur H. Dewolf, Irina A. Solopova, Daniela Morelli, Francesco Lacquaniti, Yury Ivanenko

**Affiliations:** ^1^Laboratory of Neuromotor Physiology, IRCCS Santa Lucia Foundation, Rome, Italy; ^2^Department of Pediatric Neurorehabilitation, IRCCS Santa Lucia Foundation, Rome, Italy; ^3^Centre of Space Bio-medicine and Department of Systems Medicine, University of Rome “Tor Vergata”, Rome, Italy; ^4^Laboratory of Neurobiology of Motor Control, Institute for Information Transmission Problems, Moscow, Russia

**Keywords:** cerebral palsy, abnormal development, early development of locomotion, neuromuscular pattern generation, spinal locomotor output, rehabilitation

## Abstract

The first years of life represent an important phase of maturation of the central nervous system, processing of sensory information, posture control and acquisition of the locomotor function. Cerebral palsy (CP) is the most common group of motor disorders in childhood attributed to disturbances in the fetal or infant brain, frequently resulting in impaired gait. Here we will consider various findings about functional maturation of the locomotor output in early infancy, and how much the dysfunction of gait in children with CP can be related to spinal neuronal networks vs. supraspinal dysfunction. A better knowledge about pattern generation circuitries in infancy may improve our understanding of developmental motor disorders, highlighting the necessity for regulating the functional properties of abnormally developed neuronal locomotor networks as a target for early sensorimotor rehabilitation. Various clinical approaches and advances in biotechnology are also considered that might promote acquisition of the locomotor function in infants at risk for locomotor delays.

## Introduction

The first years of life represent an extremely important phase of maturation and learning and the acquisition of bipedal locomotion is a celebrated milestone in infant development. Early injuries to developing brain may significantly affect this period of maturation and evoke impairments in the locomotor function and its delay (Rosenbaum et al., 2014). Cerebral palsy (CP) is the most common form of motor disability in childhood. It is often characterized by muscle weakness, impaired coordination of muscles and spasticity characterized by hypertonia, hyperreflexia, clonus, spasms and co-contraction (Poon and Hui-Chan, 2009). People with CP have a diversity of symptoms and severity and CP is sometimes accompanied by other disorders such as cognitive dysfunction, epilepsy, deficits in vision, speech (Christensen et al., 2014; Rosenbaum et al., 2014). Gait abnormalities represent essential concern. Indeed, about seventy percent of children with CP are able to walk though they experience problems with walking (from minimal disability to the need of walking aids), while the others require a wheelchair (Hutton and Pharoah, 2002), and life expectancy is related to the degree of impairments. This topic has broad appeal due to the general interest in the evolution of locomotion, interaction between developing spinal and supraspinal pattern generation circuitries, potential broad impact of early sensorimotor disorders, as well as its implications for understanding the basic physiological mechanisms involved.

Understanding mechanisms of early development and learning are also the basis for designing rehabilitation strategies and interventions for infants at risk for locomotor delays. We will not discuss here all aspects of impairments in the function due to CP. Instead, we will focus on motor disability in CP and gait dysfunction in particular. While the spinal pattern generation circuitry and stepping-like movements are present at birth, the locomotor behavior and the spatiotemporal structure of the motor patterns in infants undergo substantial maturation (Forssberg, 1985; Thelen and Cooke, 1987; Lacquaniti et al., 2012a; Yang et al., 2015). In the first sections, we will consider the functional and structural consequences of early injuries to developing motor regions of the brain, including pattern generation circuitry, forms of early locomotor behavior, the critical role of balance demands and sensorimotor integration, with a particular emphasis on the first years of life. We will also argue that interventions may be more efficacious if they promote quadrupedal locomotion and posture in the early months of life, and training to enhance stepping. Finally, we will consider physical therapy interventions, recent advances in biotechnology and neuromodulation of the locomotor circuitry that might promote early motor recovery in children with CP.

## Gait Impairments in CP

Detailed descriptions of gait impairments in cerebral palsy have been reported in numerous studies (Rethlefsen et al., 2017). Despite heterogeneity of symptoms and brain damage, there are typical gait abnormalities and frequent clinical problems, such as foot drop and toe walking in children with cerebral palsy. They show difficulties in developing the major features of adult gait, ankle plantarflexion with hip extension at the end of stance, increased co-activation of the leg muscles, low activation of the calf muscles, impaired ability of tibialis anterior to dorsiflex the ankle, maturation of the spinal locomotor output, and enhanced short latency proprioceptive reflexes (Berger et al., 1982, 1984; Leonard et al., 1991; Berger and Adolph, 2007; Cappellini et al., 2016).

**FIGURE 1:**
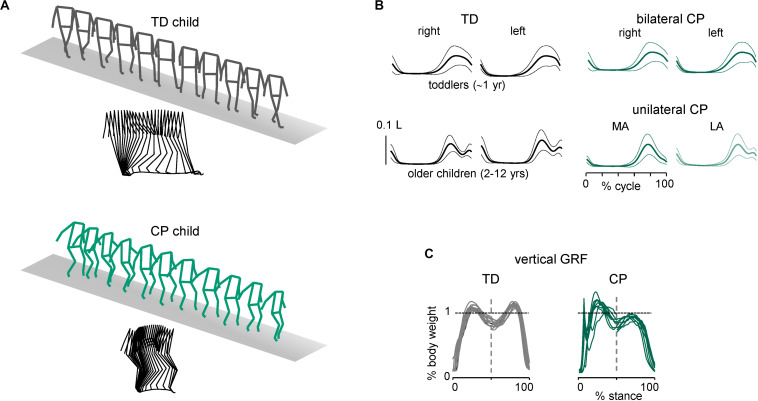
Distinctive features of ground reaction forces and foot trajectory control during walking in children with cerebral palsy. **(A)** Stick diagrams (both 3D and sagittal) of 1 stride in one TD child (5.7 years) and one child with bilateral CP (5.4 years). Note typical two peaked profile of vertical hip position during one stride in TD child (pendulum mechanism of walking) and variable pattern in child with CP. **(B)** Ensemble-averaged (across subjects, ±SD) vertical foot movements (normalized by the limb length L). MA, most affected; LA, least affected side. **(C)** Examples of vertical ground reaction forces in one TD (11.8 years) child and one representative child with bilateral CP (6.1 years). The data from several strides were superimposed (adapted from Cappellini et al., 2016).

Some characteristic features of gait are illustrated in Figure 1. In line with the general hypothesis of delayed maturation (Forssberg, 1999), many idiosyncratic features of gait in older children with CP resemble those in typically developing (TD) children at the onset of independent walking (Cappellini et al., 2016), for instance, the prominent single-peak foot lift during swing and disordered vertical hip displacements. Indeed, in addition to gait instability and slower speeds (Figure 1A), the adult-like stereotyped, two-peaked trajectory of the foot with minimal toe clearance at mid-swing representing the result of a safe, accurate endpoint control (Bernstein, 1967; Winter, 1992; Ivanenko et al., 2002) is lacking in children with CP (Figure 1B). Instead, a single-peaked foot lift is observed across all sampled ages in children with bilateral CP and on the most affected (MA) side in children with unilateral CP, typical for TD toddlers (Figure 1B). The vertical ground reaction forces often showed a decreased second peak in late stance in CP (Figure 1B), consistent with weak plantarflexion at the end of stance (Williams et al., 2011; Cappellini et al., 2016). Disordered vertical hip displacements and a lack of the gravity-related pendulum mechanism of walking in both TD toddlers (Ivanenko et al., 2004) and children with CP (Cappellini et al., 2016; Zollinger et al., 2016) are consistent with a reduced capacity in absorbing and decelerating the speed of the center of mass and in decreasing the walking energy cost.

Children with CP may develop other motor dysfunctions due to impaired corticospinal interactions, including dystonia, muscle contractures, lack of coordination (Crenna, 1998; Gormley, 2001), weak and often atrophic muscles, increased passive musculotendinous stiffness, changes in the structure of muscle fibers and connective tissue (Willerslev-Olsen et al., 2013; Mathewson and Lieber, 2015; Lieber and Fridén, 2019), so that biomechanical and histopathological changes are also contributing factors to gait abnormalities in CP (Hanson and Jones, 1989; Sutherland and Davids, 1993).

Finally, in children with disorders of the central nervous system, upper limb function is often impaired, which affects interlimb coordination and coordinative stability of limb pairs during gait. Children with CP may rely on “guard” arm postures, especially on the least affected side, as a compensation strategy to maintain balance comparable to newly walking toddlers (Meyns et al., 2012, 2016). Both less affected and more affected sides demonstrate substantially altered arm postures and movements in children with unilateral CP, associated with spasticity, balance control and other contributing factors. Given that human bipedal walking shares many features with that in quadrupeds, including similar regulation and coordination of upper and lower limb movements by central pattern generators and sensory feedback (Zehr and Duysens, 2004; Sylos-Labini et al., 2014; Solopova et al., 2016), lost or compromised arm movements in children with CP support the idea of including appropriate arm activity as a component of gait training after neurotrauma (Zehr et al., 2016; Bleyenheuft et al., 2017; Sidiropoulos et al., 2019). Thus, assessing upper limb function comprehensively is also important for planning and evaluating neurorehabilitative interventions.

## Impaired Corticospinal Pathways in CP

The control of human locomotion involves multiple neural networks including sensory, supraspinal (motor cortex, basal ganglia, thalamus, cerebellum), and spinal pattern generators signals (Grillner and El Manira, 2020). Furthermore, in contrast with many mammals, humans start to walk relatively late (Garwicz et al., 2009), and a prolonged developmental timescale can be related to postural challenges of bipedal gait, a large brain and its high rate of growth (Leigh, 2004; Kaas, 2005; Dehorter et al., 2012), and more intensive cortical participation in human locomotion than in animals (Capaday, 2002; Yang and Gorassini, 2006). Biomechanical factors, such as very slow muscle fibers at birth and even in older children (Denny-Brown, 1929; Buller et al., 1960; Dayanidhi et al., 2013), shape and soft tissues of the child’s foot sole (Maier, 1961; Gould et al., 1989; Bertsch et al., 2004), lack of extensor strength due to immature muscle cells, etc., also play a role in locomotor development and the reasons human infants do not walk sooner and do not express mature patterns (Thelen, 1995; Adolph et al., 2018; Dewolf et al., 2020). Motor problems in CP are associated with damage to motor pathways from the brain, including the corticospinal tract (CST). Importantly, the formation of specific circuits or the excitatory-inhibitory balance within them are more susceptible to damage at certain times in development in both humans and animal models of developmental motor disorders (Cavarsan et al., 2019). In particular, while reticulospinal projections from the brainstem are the first to arrive in the spinal cord followed by other tracts (Kudo et al., 1993; Sundström et al., 1993; Perreault and Glover, 2013), the CST is the last to arrive in the spinal cord (at ∼30 post-gestational week) and estimated critical period for its maturation is between few months and 2 years based on a period of CST myelination (Yakovlev and Lecours, 1967; Martin, 2005; Yeo et al., 2014). Neuroimaging methods can confirm and quantify impairments in the CST (Nemanich et al., 2019; Papadelis et al., 2019). Given that CST projection activities significantly shape the spinal cord motor function (Eyre et al., 2001), neuronal activity appear to be essential during the critical period for the normal development of the motor circuits (Yang et al., 2013).

One way to probe the development of functional corticospinal connectivity is to estimate the oscillatory drive of the motor cortex to the spinal cord using coherence analysis of MEG/EEG and EMG signals (Ritterband-Rosenbaum et al., 2017). For instance, beta and gamma frequency drive to the motor pool can be accessed through the surface EMG by evaluating coherence and synchronization of motor units within and between muscles. Beta frequency oscillations (15–35 Hz), which are coherent with similar frequencies in corticomuscular coherence in healthy adults (Salenius et al., 1996; Mima and Hallett, 1999), have been shown to be impaired in CNS lesions (Hansen et al., 2005; Nielsen et al., 2008). Thus, coherence and synchrony between EMGs are dependent on intact central motor pathways and these features may serve as physiological markers of impaired supraspinal control of gait (Hansen et al., 2005).

**FIGURE 2:**
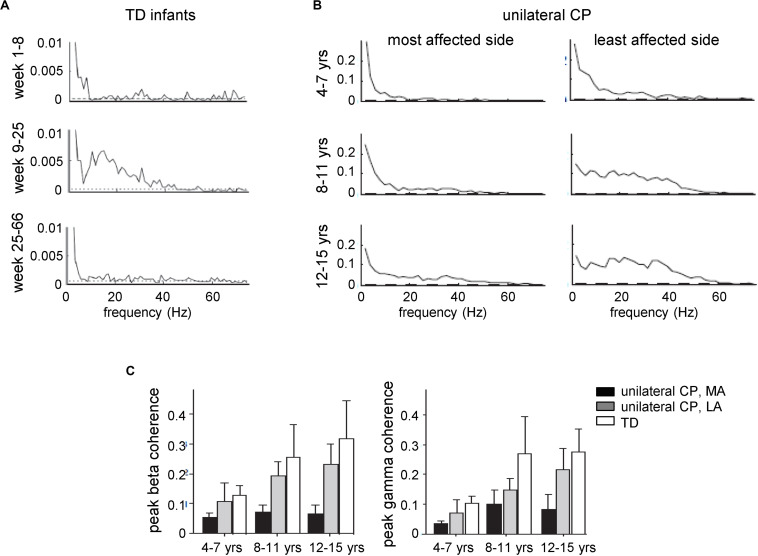
Development of central common drive to a leg muscle. EMG traces were obtained from electrodes placed at the proximal and distal part of the tibialis anterior muscle. Coherence estimates provide a measure of the fraction of the activity in one surface EMG signal at any given frequency that can be predicted by the activity in the second surface EMG signal, reflecting the strength of common rhythmic synaptic inputs distributed across the motoneuron pool. **(A)** EMG–EMG pooled coherence at different frequencies for the three age groups given by the corrected age: 1–8, 9–25, and 25–66 weeks. The dashed lines indicate the 95% confidence levels for the pooled data (adapted from Ritterband-Rosenbaum et al., 2017 with permission). **(B)** Pooled estimates of coherence from all subjects with unilateral CP for the most and least affected sides for three different age groups: 4–7, 8–11, and 12–15 years. **(C)** Peak beta-band and gamma-band coherences in CP (MA, most affected; LA, least affected side) and TD children across the three different age groups. Error bars denote 95% confidence intervals [panels **(B,C)** adapted from Petersen et al., 2013 with permission].

This method has also been used to evaluate developmental changes of functional corticospinal connectivity. For instance, recent data suggest that the corticospinal drive to muscles shows significant developmental changes with an increase in functional coupling in infants aged 9–25 weeks (Figure 2A; Ritterband-Rosenbaum et al., 2017), a sensitive period which coincides with the developmental period of normal fidgety movements in TD infants, noticeable manifestation of muscle reactions and self-organization of neural circuits (Blankenship and Feller, 2010; Hadders-Algra, 2018; Solopova et al., 2019). The coherence and synchrony between EMGs undergo developmental increases in late childhood (Figure 2B, TD children). In children with CP, there is a frequent problem of foot drop during gait associated with impaired control of the ankle dorsiflexors and reflected also in impaired tibialis anterior EMG-EMG coherence in the beta and gamma frequency bands on the most affected side, as well as lack of age-related increase of coherence (Figure 2; Petersen et al., 2013). Furthermore, toe walking in children with CP appears to be controlled differently from voluntary toe walking in typically developing children and is accompanied by differences in motor unit synchronization and coherence between antagonist EMGs (Lorentzen et al., 2019). Interestingly, 4 weeks of daily intensive treadmill training with an incline in children with CP may improve the control of the ankle joint (number and amplitude of toe lifts in the swing phase) and evoke plastic changes in the corticospinal tract associated with increased beta and gamma oscillatory drive to motoneurons (Willerslev-Olsen et al., 2015).

## Neuromuscular Generation and Maturation of Locomotor Circuitry in Early Infancy

While the above-mentioned assessments of the functional corticospinal connectivity provide important information about output of the motor cortex and its transmission to the spinal cord, one should keep in mind that these measurements are nevertheless limited in their ability to assess the actual state of the spinal locomotor circuitry and its impairment in CP. Indeed, whereas subcortical and cortical structures coordinate locomotor responses, especially when gait is made more difficult by demanding external conditions or postural instability, the basic neural control mechanism is largely governed by spinal pattern generators (Kiehn, 2016; Minassian et al., 2017; Gill et al., 2018; Grillner and El Manira, 2020). Using electrophysiological, pharmacological, or neuroanatomical approaches in invertebrates and vertebrates, the identification of the spinal interneurons and investigation of the locomotor circuitry provided important insights into how these functional circuits are formed during development. In particular, such studies showed considerable reorganization of spinal circuitry and the involvement of new circuitry during early development of locomotion (Vinay et al., 2002; Rauscent et al., 2006; Chakrabarty et al., 2009; Fetcho and McLean, 2010; Currie and Sillar, 2018). Therefore, even though the primary deficit in children with CP originates from the damage to the brain, a large part of the locomotor dysfunction might be attributable to the impaired state of the developing spinal circuitry, which has been somewhat overlooked.

An essential aspect of damage to developing brain is a risk of substantial or even irreversible changes in the state of the locomotor network during early development and critical developmental windows in particular. Moreover, if the state of the spinal circuitry is impaired, it should be controlled differently by descending motor pathways, which in turn would enhance the reorganization and involvement of the supraspinal structures to compensate for these abnormalities. These reciprocal spinal-supraspinal compensatory mechanisms create a risk of irreversible changes in the state of locomotor circuitry during early development, especially during critical developmental windows (Hadders-Algra, 2004; Yang et al., 2013; Friel et al., 2014; Cappellini et al., 2016).

What are indicators of the spinal cord involvement in CP? First, although it has been argued that the proximity of the spinal circuitry to the outer world may demand a more rigid organization compared to the highly flexible cortical circuits (Christiansen et al., 2017), this statement is valid only to some extent and unlikely for the developing spinal cord. Definitely, the spinal cord is not a simple relay structure for communication between central structures and skeletal musculature but is flexible (Heng and de Leon, 2007), capable of performing coordinate transformations (Fukson et al., 1980; Windhorst, 1996a; Poppele and Bosco, 2003), synapse daily turnover, cell death and atrophy after a spinal cord injury (Dietz and Müller, 2004; Gazula et al., 2004) or after brain damage (Drobyshevsky and Quinlan, 2017). In humans, examination of spinal neuronal circuitries is difficult to perform by non-invasive methods though some structural changes were documented. For instance, postmortem examination of children with CP showed abnormalities in the rostral segments of the spinal cord (Levchenkova and Semenova, 2012), while magnetic resonance imaging of the spinal cord in the subjects with spastic bilateral CP showed a reduced white matter crosssectional area at C6/C7 and T10/T11 segments (Noble, 2014). Early corticospinal lesion at the spinal level in humans also affects the immature spinal cord and gait maturation (Dan et al., 2004). As far as it concerns the mechanisms of early motor dysfunctions in CP, animal studies convincingly show that injury to the supraspinal systems or removing descending input severely disrupts spinal cord neuromodulation and the postnatal development of spinal circuits (Clowry, 2007; Friel et al., 2014; Smith et al., 2017; Jiang et al., 2018). The spinal interneurons mature in common with the CST connections (Chakrabarty et al., 2009) and extensively in the early period (possibly equivalent to ages 3–5 months in human infants), suggesting that if that window closes, full recovery is not possible (Friel et al., 2014). Furthermore, unilateral CST inactivation produces not only contralateral but also ipsilateral effects on the developing spinal circuitry, due to both sparse ipsilateral terminations and indirect ipsilateral influences at multiple levels of the CNS, reflecting the balanced contributions from the motor cortex on each side, rather than overwhelmingly from the contralateral side (Friel and Martin, 2007). Descending pathways also regulate spontaneous activity, which is likely a major trigger for early maturation of lumbar locomotor networks (Vinay et al., 2002).

Second, most synapses in the spinal cord are inhibitory (Levine et al., 2014) and contribute to network stability, preparation of an appropriate state of spinal circuitries to accommodate a specific supraspinal command (since the same interneurons and motoneurons participate in a wide range of movements and synergistic actions) and avoiding an excessive motor reaction (Windhorst, 1996b). However, in individuals with CP, damaging cortico-, rubro-, reticulo-, and vestibulo-spinal glutamatergic projections to the spinal cord through spinal inhibitory interneurons (Jankowska et al., 1976) can reduce inhibitory tone in the spinal cord and contribute to hypertonia (Sanger, 2003; Deon and Gaebler-Spira, 2010). The excitatory-inhibitory misbalance in the spinal circuitry in persons with CP is manifested by enhanced segmental reflexes with abnormal radiation of stretch reflexes to other muscles including the lack of the development of reciprocal inhibition of antagonist muscles (Berger et al., 1982, 1984; Myklebust et al., 1982; Myklebust, 1990), and the greater the imbalance the more severe the motor disorders (Condliffe et al., 2016).

Third, neuromodulation of the physiological state of the spinal cord is known to affect locomotor performance (Ivanenko et al., 2017; Gill et al., 2018). For instance, the locomotor function can be improved in children with CP using transcutaneous spinal cord stimulation during gait training (Solopova et al., 2017). It is also worth mentioning that these promising findings have been obtained in relatively older children (7–11 years), when substantial spinal abnormality induced by perinatal brain damage was already developed, and they need to be explored further to assess more comprehensively the more responsive neuromechanical characteristics and age-effect of such locomotor improvements. In addition to influences on locomotor function (Solopova et al., 2017), high-frequency spinal cord stimulation may reduce spasticity in children with CP (Shabalov et al., 2006; Dekopov et al., 2015).

To end with, the final neural output of spinal locomotor circuitry is represented by the spatiotemporal modulation of alpha-motoneuron (MN) activity, which can be assessed by mapping the activity patterns from a large number of simultaneously recorded muscles onto the anatomical rostrocaudal location of the MN pools in the spinal cord (Yakovenko et al., 2002; Ivanenko et al., 2013; Wenger et al., 2016), and by decomposing the coordinated muscle activation profiles into a small set of common factors as a means to look backward from the periphery to the CNS (Davis and Vaughan, 1993; Lacquaniti et al., 2012b). There are now several studies that evaluated the spatiotemporal organization of the spinal locomotor output in CP (Steele et al., 2015, 2019; Tang et al., 2015; Cappellini et al., 2016; Shuman et al., 2016, 2017, 2018, 2019a,b; Hashiguchi et al., 2018; Kim et al., 2018; Booth et al., 2019; Yu et al., 2019; Falisse et al., 2020; Pitto et al., 2020; Short et al., 2020).

**FIGURE 3:**
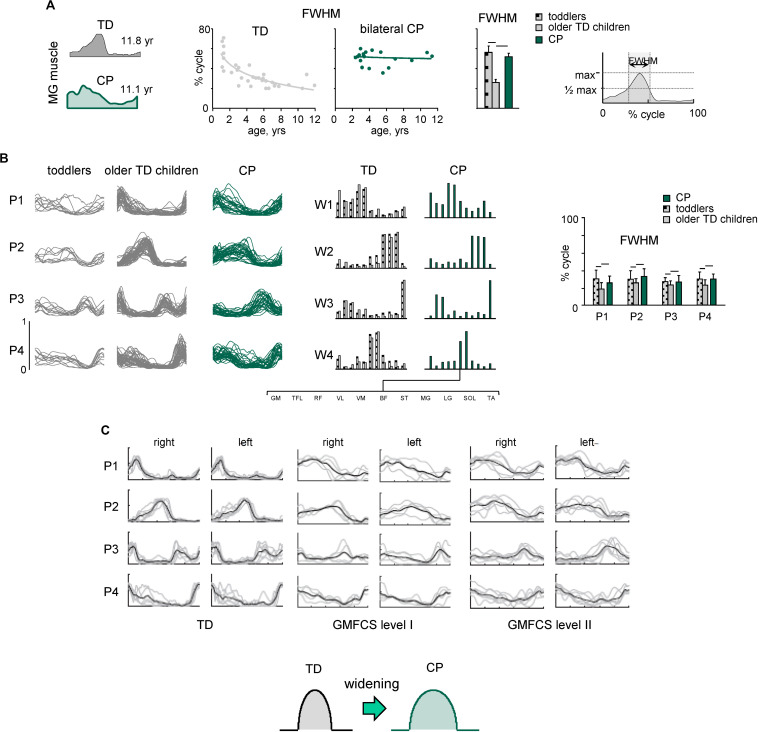
Spatiotemporal organization of muscle activity patterns during walking. **(A)** Developmental trend for the duration of muscle (MG, medial gastrocnemius) activity. From left to right: examples of MG activity in one TD child and one child with bilateral CP, duration of MG activity (FWHM, full width at half maximum, see right panel) as a function of age (continuous lines represent exponential fittings), and averaged across children [horizontal lines denote significant differences compared with older TD children (2–12 years)]. **(B)** Statistical analysis of EMG patterns: basic activation patterns P1–P4 (each curve represents the pattern for an individual child) and corresponding weights W1–W4 (muscle synergies). Right panel – mean (+SD) FWHM of consistent basic activation patterns (P1–P4). Adapted from Cappellini et al. (2016). **(C)** Basic activation patterns (for the right and left sides) in TD children and children with bilateral CP at Gross Motor Function Classification System (GMFCS) levels I and II (adapted from Yu et al., 2019 with permission). Note significantly wider patterns in CP, independent of GMFCS level.

**FIGURE 4:**
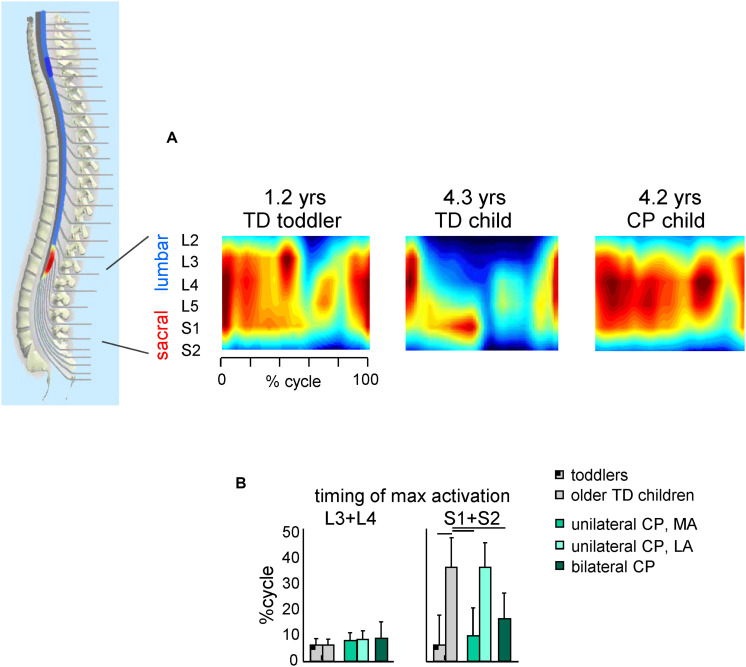
Spatiotemporal maps of motoneuron activity of the lumbosacral enlargement in TD children and children with CP. **(A)** Examples of segmental output in one TD toddler (1.2 years), one TD older child (4.3 years) and one child with CP (4.2 years) (adapted from Cappellini et al., 2016). **(B)** Timing (+SD) of maximum activation of lumbar (L3 + L4) and sacral (S1 + S2) segments. Lines over bars denote significant differences compared with older TD children.

Figures 3, 4 illustrate typical features of spinal locomotor output impairments in CP. TD children show a progressive reduction of EMG burst durations with increasing age (Figure 3A) likely reflecting an essential developmental aspect of muscular control optimization. This might be important for coordination of locomotion with voluntary movements, which requires a precise coordination of activation timings of the locomotor and voluntary motor programs (Ivanenko et al., 2005), and for optimization of the energetic cost of walking. For the assessment of motor coordination, one may test a modular approach for neuromuscular control providing information about temporal patterns of muscle activation shared by different muscles along with corresponding muscle synergies (Davis and Vaughan, 1993; Lacquaniti et al., 2012b). Such factorization of the EMG signals revealed a comparable structure of the motor output in children with CP and TD children, but significantly wider temporal activation patterns in children with CP, resembling the patterns of much younger TD infants (Figure 3B; Cappellini et al., 2016). Reduction of dimensionality (the smaller number of muscle synergies) found in some previous studies (e.g., Steele et al., 2015; Tang et al., 2015; Shuman et al., 2016) may depend on the criterion used to define the minimum number of synergies (Hug et al., 2012; Russo et al., 2014) and/or the limited number of recorded muscles (Steele et al., 2013; Zelik et al., 2014; Damiano, 2015). Nevertheless, the observed phenomenon of widening (Figure 3B) does not depend on the exact number of modules retained by the specific non-negative matrix factorization procedure (Martino et al., 2015), was confirmed in other studies as well, and seems to be a characteristic feature of CP gait. Furthermore, wider basic muscle activity patterns in CP were observed independent of the GMFCS level (Figure 3C; Yu et al., 2019). Thus, locomotor patterns of older children with cerebral palsy show lack of maturation and similarity of the early stages of gait development in healthy children.

A similar picture emerges when considering the spatiotemporal maps of alpha-motoneuron activation (Figure 4). The spinal maps of motor pool activation can be estimated by mapping EMG activity of a large number of simultaneously recorded muscles onto the anatomical rostrocaudal location of the MN pools under the assumption that the rectified EMG provides an indirect measure of the net firing of MNs of that muscle in the spinal cord (Yakovenko et al., 2002; Ivanenko et al., 2013). TD children show a gradual reorganization of the spatiotemporal MN output with increasing age (Ivanenko et al., 2013; Dewolf et al., 2020), consisting in more narrow loci of MN activity and a progressive shift of the timing of maximum activation of sacral segments toward later stance (Figure 4B, right panel). By contrast, this developmental trend in children with CP is lacking (on both sides for children with bilateral CP and the affected side for children with unilateral CP). Therefore, children with CP show very limited age-related changes of muscle activity pattern durations and motoneuron output (Cappellini et al., 2016), consistent with the idea that early injuries to developing brain substantially affect the maturation of the spinal locomotor output.

In sum, how intrinsic spinal locomotor circuits are remodeled after a perinatal brain injury needs to be better understood since they play a key role in locomotor dysfunction in CP and in developing locomotor neuromuscular pattern generation in general, taking into consideration a substantial ongoing reorganization of the locomotor output in TD infants during the first year of life (Dominici et al., 2011; Ivanenko et al., 2013; Sylos-Labini et al., 2020). Also, the efficacy to repair supraspinal (CST) connections to the spinal cord is strongly reduced after the critical period and is insufficient to restore significant function unless promoted (Friel et al., 2014). This suggests the necessity for early central pattern generator-modulating therapies and early gait rehabilitation in children with CP to assist in the normal development of the spinal motor circuits and enhancing walking (Yang et al., 2013; Cappellini et al., 2016; Hurd et al., 2017).

## Adaptive Gait Control in CP

Locomotor movements must be accommodated to different environments and directions of progression. The ability to adapt is of particular interest in the context of cerebral dysfunction, since the control of adaptive locomotion may involve accurate foot placements, their visual guidance, changes in the coordination, greater balance control, anticipatory locomotor adjustments, and thus require larger cortical involvement. Whereas the impairments of standard forward ‘steady state’ gait on a flat surface have been extensively investigated in children with CP, the neural mechanisms of the adaptive locomotor behavior have been studied to a lesser extent, even though difficulties in performing complex locomotor movements (walking on inclines, uneven terrain, in crowded area, climbing stairs) are included in the GMFM (Gross Motor Function Measure) assessment in persons with CP. Below we consider some examples of such movements supporting the idea that complex locomotor movements can be used for more comprehensive diagnosis of CP as well as for gait rehabilitation.

**FIGURE 5:**
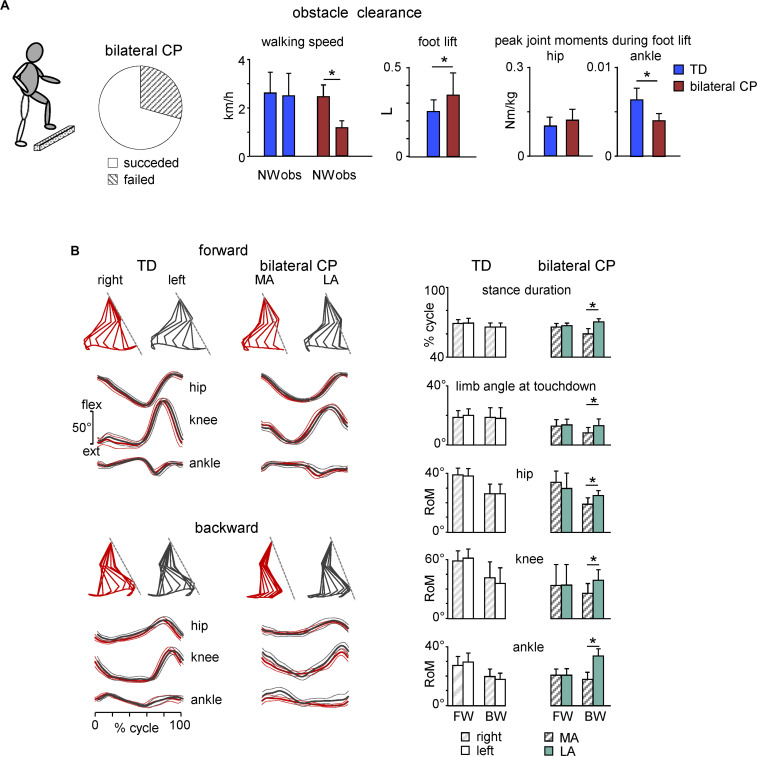
Adaptive locomotion in children with CP. **(A)** Obstacle task performance in children with bilateral CP. From left to right: pie chart showing the percentage of trials for children with succeeded and failed obstacle clearance, mean (+SD) walking speed for successful trials, foot lift and flexor peak hip and ankle muscle moments during trailing limb elevation. Asterisks denote significant side differences. Adapted from Cappellini et al. (2020). **(B)** Kinematics of forward and backward walking in TD and children with bilateral CP. Left panels: examples of forward gait kinematics in one TD child (5.7 years) and one child with CP (6.1 years) during forward and backward walking. From top to bottom: stick diagrams of the swing phase of a single step for both legs (relative to the instantaneous hip position), and ensemble-averaged (across all strides, ±SD) hip, knee, and ankle joint angles (red corresponds to the most affected side in CP). Dashed lines in stick diagrams correspond to the orientation of the left (in TD) and least affected (in CP) limb during touchdown. Right panels: comparison of gait parameters and ranges of angular motion (+SD) for both legs in TD and children with bilateral CP during forward (FW) and backward (BW) walking. MA, most affected; LA, least affected side. Adapted from Cappellini et al. (2018).

Locomotion rarely occurs on a flat surface and we often encounter obstacles in our pathway. In general, children with CP have difficulties in clearing an obstacle, being slower in approach and crossing speed along with unsteadiness of gait and balance adaptations of the trunk control (Law and Webb, 2005; Malone et al., 2016). For instance, in a recent study (Cappellini et al., 2020) we showed that about 30% of children with bilateral CP failed to perform the task (they stopped before the obstacle, performed lateral obstacle avoidance, stumbled or stepped onto the obstacle). Interestingly, they had mostly posterior lesions of the brain (Cappellini et al., 2020), in relation to their deficits in the anticipatory visuomotor control and important role of parietal lobe activity in visually planning gait adaptations (Drew et al., 2008; Lajoie et al., 2010; Drew and Marigold, 2015). Remaining children with CP (∼70%), who succeeded with obstacle clearance, performed the task significantly slower than age-matched TD children, demonstrating a high foot lift of the trailing (unseen) limb, smaller range of motion and muscle moments of the distal (ankle) joint (Figure 5A, left panels), and limited adaptation of task-relevant activity of hamstring muscles timed to the voluntary task of foot lift over the obstacle (Cappellini et al., 2020). Thus, impaired task performance in children with CP may reflect basic developmental deficits in the adaptable control of gait when the locomotor task is superimposed with the voluntary movement, suggesting that gait rehabilitation strategies should involve tasks performed in challenging environments to enhance the functional capacity of gait controllers.

Backward walking (BW) is another example of adaptive locomotor behavior. It has been argued that BW uses the same rhythm circuitry as forward walking (FW) but involves additional specialized control circuits (Hoogkamer et al., 2014). BW is also a beneficial physical activity used in the rehabilitation of children with CP to improve walking abilities, strengthen RF and TA muscles, as well as augmenting hip extension and knee flexion with ankle dorsiflexion (Kim et al., 2016; Abdel-Aziem and El-Basatiny, 2017; Hösl et al., 2018; Elnahhas et al., 2019). Furthermore, BW highlights prominent gait asymmetries in children with CP and thus may give a more comprehensive assessment of the gait pathology (Cappellini et al., 2018). In particular, gait asymmetries, which were not evident during FW in children with bilateral CP, became evident during BW (Figure 5B). Accordingly, the most affected side in bilateral cerebral palsy can be defined based on the limb that show shorter stance duration during BW. The definition of unilateral cerebral palsy is usually not etiologic but functional (Bax et al., 2005; Rosenbaum et al., 2014), as a neuromuscular disorder that involves one half of the body (most affected side). The reason for the lack of asymmetry during FW in children with bilateral CP might be explained by the fact that the diagnosis of asymmetry is determined by clinical observation (e.g., the side on which the leg has the highest spasticity measure), and to our knowledge there is no valid criterion based on instrumented gait analysis to distinguish between asymmetric and symmetric children with bilateral CP. BW may also be more asymmetric because it is a less practiced form of gait than FW. Walking asymmetry can be problematic for many reasons and is increasingly measured and used as an important marker of gait recovery after stroke (Patterson et al., 2010; Wonsetler and Bowden, 2017). Spatiotemporal asymmetry assessments during BW in CP might reflect an impaired state and/or descending control of the spinal locomotor circuitry and can be used to help the diagnosis of the most affected side and as complementary markers of gait recovery.

To sum up, early injuries to developing brain affect both normal walking and other forms of locomotor behavior: complex locomotor movements (Law and Webb, 2005; Dixon et al., 2016; Mawase et al., 2016; Lewerenz et al., 2019; Cappellini et al., 2020), running (Böhm and Döderlein, 2012), weighting of legs (Bulea et al., 2017), backward walking (Cappellini et al., 2018), and even earlier locomotor movements such as crawling in infants with developmental delay (Xiong et al., 2018; Li et al., 2019). Current interventions are being developed that emphasize including more complex and voluntary locomotion in gait rehabilitation of children with CP. For instance, intensive training of walking on the inclined surface represents a promising protocol aimed at improving the control of the ankle joint and foot drop in CP (Willerslev-Olsen et al., 2015), while the standardized walking obstacle course was included as a part of movement therapy (Malone et al., 2016). Given that the support surface and external objects are included in the locomotor body scheme and its development (Dominici et al., 2010; Pearson and Gramlich, 2010; Ivanenko et al., 2011), navigating complex terrain, e.g., using the “magic carpet,” can further enhance spatial representation and generation of locomotor trajectories in CP (Berthoz and Zaoui, 2015; Belmonti et al., 2016). Thus, the best possible understanding of the impaired control of compound locomotor movements and their development is relevant for the ongoing work on improvement of the locomotor function in early childhood in persons with CP.

## Early Interventions to Promote the Locomotor Function in Infants With CP

The development of efficient and independent walking is an important therapeutic goal for children with CP (Willoughby et al., 2009; Smania et al., 2011; Degelean et al., 2012; Drużbicki et al., 2013; Willerslev-Olsen et al., 2015; Graham et al., 2016; Lerner et al., 2017). This may include advances in biotechnology to unveil new information about the impaired locomotor output or infant general movements for the early diagnosis of CP (Zhu et al., 2015; Redd et al., 2019; Airaksinen et al., 2020; Sylos-Labini et al., 2020), to develop central pattern generator-modulating therapies (Solopova et al., 2017) and to enhance walking. For example, initially shown to be effective for mammalian gait retraining (e.g., Barbeau and Rossignol, 1987; van den Brand et al., 2015; von Zitzewitz et al., 2016), a therapeutic intervention for gait retraining with partial body weight support using a harness system (McNevin et al., 2000) or water immersion (Oliveira et al., 2014) may improve walking capacity in children with CP (Day et al., 2004; Azizi et al., 2017). Given a positive effect of repetitive locomotor exercise on gait characteristics in CP (Smania et al., 2011; Willerslev-Olsen et al., 2015), also with the use of wearable exoskeleton (Lerner et al., 2017), the rehabilitative protocol may further focus on improving the locomotor output, e.g., by providing a feedback on specific features of the spinal locomotor output (Figures 3, 4) or implementing gait training program with real-time feedback of the body’s center-of-mass vertical displacement to restore the pendulum mechanism and decrease the walking energy cost (Massaad et al., 2010). Such approaches may be complementary to current concepts in rehabilitation of gait in children with CP. In addition, combining gait training with spinal cord neuromodulation may also improve locomotor function (Solopova et al., 2017). In addition, given that the muscles are weak and often quite atrophic in children with CP, resulting in significantly reduced volumes in leg muscles and in bone changes (Barrett and Lichtwark, 2010; Moreau et al., 2010; Oberhofer et al., 2010; Willerslev-Olsen et al., 2013; Noble et al., 2014; Handsfield et al., 2016), interventions increasing muscle length or strength can also improve gait. Early recognition of progressive deformity in the muscles and joints of the lower extremity and the spine in children with CP may allow timely treatment and prevention of irreversible changes (Morrell et al., 2002; Barber et al., 2011; Handsfield et al., 2016). Nevertheless, the reported gait recovery or power to find significant results still remains often limited (Valentín-Gudiol et al., 2017). Furthermore, in the great majority of studies, the CP participants benefited from locomotor training after the age of 3–5 years, keeping also in mind the delayed onset of independent walking in many infants with CP.

Frequent treatment for the lower limbs in young children with CP is more passive, typically including stretching, an ankle-foot orthosis for the affected leg (Wingstrand et al., 2014), and botulinum toxin A injections to reduce the abnormal muscle tone (Koman et al., 2003). Given critical developmental periods for maturation of the locomotor networks and corticospinal connectivity (see above section “*Neuromuscular Generation and Maturation of Locomotor Circuitry in Early Infancy*”), the key missing element in the majority of studies focusing on neurodevelopmental treatment - intensive child-initiated motor activity (Hurd et al., 2017). However, investigations focusing on early therapy of the lower limbs and the locomotor function are sparse (Richards et al., 1997; Campbell et al., 2012; Prosser et al., 2012; Hurd et al., 2017; Kolobe and Fagg, 2019).

Based on knowledge of neuroplasticity and the idea of critical developmental windows (Yang et al., 2013; Friel et al., 2014; Hadders-Algra, 2014; Reid et al., 2015; Cappellini et al., 2016; Hurd et al., 2017; Williams et al., 2017), the potential impact of initiating training at an earlier age is also an important consideration for clinicians working with children with CP. It is also worth noting available evidence for early accurate diagnosis of cerebral palsy that now can be made before 6 months’ corrected age (Novak et al., 2017). Taking advantage of newly available biotechnology for pediatric rehabilitation, training in the sensitive period for maturation would help to optimize infant motor and cognitive plasticity and enhance more effectively their locomotor function, that we briefly consider below.

**FIGURE 6:**
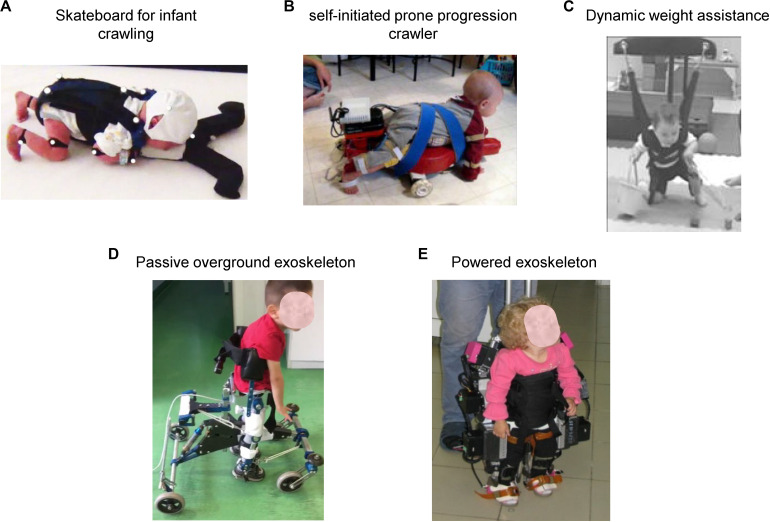
Physical therapy interventions that may promote early locomotor movements and enhance stepping for infants at risk for locomotor delays. **(A)** Pediatric skateboard for entraining quadrupedal locomotion (crawling) in infants in early months of life [reproduced from Forma et al. (2019) with permission]. **(B)** Self-initiated prone progression crawler containing active components enabling the infant to push harder to produce the intended movement [reproduced from Kolobe and Fagg (2019) with permission]. **(C)** Dynamic weight assistance system allowing practice of motor skills that infants are not yet able to perform on their own (crawling, knee walking, walking, climbing, attempts to jump and falling) [reproduced from Prosser et al. (2012) with permission]. **(D)** Passive non-actuated exoskeleton (“Moonwalker” used to facilitate upright posture and stepping in young children with cerebral palsy (courtesy of Hilenia Catania). **(E)** Powered exoskeleton (“ExoAtlet Bambini”) for assisting stepping in children suffering from early neurological gait impairment (courtesy of Elena Pismennaya).

Figure 6 illustrates some recent technological assistive solutions for implementing early locomotor behavior therapy in children with CP younger than 2 years of age. For instance, Forma et al. (2019) argued that a quadrupedal organization underlying locomotor movements in humans is manifested rather early (see also La Scaleia et al., 2018), particularly apparent on the skateboard (Figure 6A), and thus early quadrupedal training may enhance interventions designed to hasten the onset of independent walking in infants with cerebral palsy and developmental delays. In the same vein, the SIPPC (self-initiated prone progression crawler) system represents an integration of robotics and sensor technologies designed to capture (recognize one of 20 different crawling-like gestures of the arms and feet) and influence movement effort as infants learn prone locomotion (Kolobe and Fagg, 2019). With this idea of early crawling, it is worth stressing the fact that upper limb retraining may induce modification of locomotor function (Delabastita et al., 2016; Meyns et al., 2017).

The potential for infants to learn new behaviors and the acquisition of early locomotor function is also important for shaping and normal maturation of sensorimotor integration and psychological development (Anderson et al., 2013, 2016, 2019). Therapy utilizing novel dynamic weight assistance technology (Prosser et al., 2012; Figure 6C) may allow practice of motor skills that children are not yet able to perform on their own (crawling, knee walking, walking, climbing, attempts to jump and falling). A passive non-actuated exoskeleton provides further opportunity to develop the child’s potential for independent movement (“Moonwalker” and “NF-Walker^®^” available in the market, Figure 6D). It can also be modified using the actuated robotic aid by means of artificial muscles (Smania et al., 2012) and represents an individually adjustable device with a high amount of postural control, which assists children with severe gait impairment to attain independent mobility in standing position (Kuenzle and Brunner, 2009). Finally, a recently developed powered exoskeleton (“ExoAtlet Bambini,” Figure 6E)^1^ is able to provide assistance for stepping and help young children (∼2 years) to learn how to walk. Stimulation or early training of the locomotor function may have a greater impact on the onset of independent walking for children with development disorders and have the potential to alter the trajectory of motor development in CP (Prosser et al., 2012).

Research is also required to explore neural changes in response to training, especially given the capacity for change in developing nervous systems. In this respect, better understanding of early remodeling of intrinsic locomotor circuits after a perinatal brain injury is warranted to evaluate and develop successful strategies for early interventions in infants at risk of developmental delays. Studies using animal models of cerebral palsy could further advance our ability to treat and cure a variety of conditions (e.g., using medication and neuromodulation of neuronal circuits, Cavarsan et al., 2019), ameliorate motor symptoms, facilitate a more basic mechanistic understanding of the neurobiological underpinnings of neuroplasticity of cerebral palsy and develop early central pattern generator-modulating therapies.

## Author Contributions

All the authors made contributions in drafting the manuscript and have approved the final version.

## Conflict of Interest

The authors declare that the research was conducted in the absence of any commercial or financial relationships that could be construed as a potential conflict of interest.

## Footnotes

1 https://exoatlet.lu/bambini/

## References

[B1] Abdel-Aziem A. A. El-Basatiny H. M. (2017). Effectiveness of backward walking training on walking ability in children with hemiparetic cerebral palsy: a randomized controlled trial. *Clin. Rehabil.* 31 790–797. 10.1177/0269215516656468 27356944

[B2] Adolph K. E. Hoch J. E. Cole W. G. (2018). Development (of Walking): 15 suggestions. *Trends Cogn. Sci. (Regul. Ed.)* 22 699–711. 10.1016/j.tics.2018.05.010 30032744 PMC6145857

[B3] Airaksinen M. Räsänen O. Ilén E. Häyrinen T. Kivi A. Marchi V. (2020). Automatic posture and movement tracking of infants with wearable movement sensors. *Sci. Rep.* 10:169. 10.1038/s41598-019-56862-5 PMC6957504 31932616

[B4] Anderson D. I. Campos J. J. Witherington D. C. Dahl A. Rivera M. He M. (2013). The role of locomotion in psychological development. *Front. Psychol.* 4:440. 10.3389/fpsyg.2013.00440 23888146 PMC3719016

[B5] Anderson D. I. He M. Gutierrez P. Uchiyama I. Campos J. J. (2019). Do balance demands induce shifts in visual proprioception in crawling infants? *Front. Psychol.* 10:1388. 10.3389/fpsyg.2019.01388 31281282 PMC6595268

[B6] Anderson D. I. Kobayashi Y. Hamel K. Rivera M. Campos J. J. Barbu-Roth M. (2016). Effects of support surface and optic flow on step-like movements in pre-crawling and crawling infants. *Infant. Behav. Dev.* 42 104–110. 10.1016/j.infbeh.2015.11.005 26773774 PMC4769934

[B7] Azizi S. Marzbani H. Raminfard S. Birgani P. M. Rasooli A. H. Mirbagheri M. M. (2017). The impact of an anti-gravity treadmill (AlterG) training on walking capacity and corticospinal tract structure in children with cerebral palsy. *Conf. Proc. IEEE Eng. Med. Biol. Soc.* 2017 1150–1153. 29060079 10.1109/EMBC.2017.8037033

[B8] Barbeau H. Rossignol S. (1987). Recovery of locomotion after chronic spinalization in the adult cat. *Brain Res.* 412 84–95. 10.1016/0006-8993(87)91442-9 3607464

[B9] Barber L. Hastings-Ison T. Baker R. Barrett R. Lichtwark G. (2011). Medial gastrocnemius muscle volume and fascicle length in children aged 2 to 5 years with cerebral palsy. *Dev. Med. Child Neurol.* 53 543–548. 10.1111/j.1469-8749.2011.03913.x 21506995

[B10] Barrett R. S. Lichtwark G. A. (2010). Gross muscle morphology and structure in spastic cerebral palsy: a systematic review. *Dev. Med. Child. Neurol.* 52 794–804. 10.1111/j.1469-8749.2010.03686.x 20477832

[B11] Bax M. Goldstein M. Rosenbaum P. Leviton A. Paneth N. Dan B. (2005). Executive committee for the definition of cerebral palsy. Proposed definition and classification of cerebral palsy, April 2005. *Dev. Med. Child Neurol.* 47 571–576. 16108461 10.1017/s001216220500112x

[B12] Belmonti V. Cioni G. Berthoz A. (2016). Anticipatory control and spatial cognition in locomotion and navigation through typical development and in cerebral palsy. *Dev. Med. Child. Neurol.* 58(Suppl 4) 22–27. 10.1111/dmcn.13044 27027604

[B13] Berger S. E. Adolph K. E. (2007). Learning and development in infant locomotion. *Prog. Brain Res.* 164 237–255. 10.1016/s0079-6123(07)64013-8 17920435

[B14] Berger W. Altenmueller E. Dietz V. (1984). Normal and impaired development of children’s gait. *Hum. Neurobiol.* 3 163–170. 6480437

[B15] Berger W. Quintern J. Dietz V. (1982). Pathophysiology of gait in children with cerebral palsy. *Electroencephalogr. Clin. Neurophysiol.* 53 538–548. 6177498 10.1016/0013-4694(82)90066-9

[B16] Bernstein N. A. (1967). *The Co-Ordination and Regulation of Movements.* London: Pergamon Press.

[B17] Berthoz A. Zaoui M. (2015). New paradigms and tests for evaluating and remediating visuospatial deficits in children. *Dev. Med. Child Neurol.* 57(Suppl 2) 15–20. 10.1111/dmcn.12690 25690111

[B18] Bertsch C. Unger H. Winkelmann W. Rosenbaum D. (2004). Evaluation of early walking patterns from plantar pressure distribution measurements. First year results of 42 children. *Gait Posture* 19 235–242. 10.1016/s0966-6362(03)00064-x 15125912

[B19] Blankenship A. G. Feller M. B. (2010). Mechanisms underlying spontaneous patterned activity in developing neural circuits. *Nat. Rev. Neurosci.* 11 18–29. 10.1038/nrn2759 19953103 PMC2902252

[B20] Bleyenheuft Y. Ebner-Karestinos D. Surana B. Paradis J. Sidiropoulos A. Renders A. (2017). Intensive upper- and lower-extremity training for children with bilateral cerebral palsy: a quasi-randomized trial. *Dev. Med. Child. Neurol.* 59 625–633. 10.1111/dmcn.13379 28133725

[B21] Böhm H. Döderlein L. (2012). Gait asymmetries in children with cerebral palsy: do they deteriorate with running? *Gait Posture* 35 322–327. 10.1016/j.gaitpost.2011.10.003 22055251

[B22] Booth A. T. C. van der Krogt M. M. Harlaar J. Dominici N. Buizer A. I. (2019). Muscle synergies in response to biofeedback-driven gait adaptations in children with cerebral palsy. *Front. Physiol.* 10:1208. 10.3389/fphys.2019.01208 31611807 PMC6776587

[B23] Bulea T. C. Stanley C. J. Damiano D. L. (2017). Part 2: adaptation of gait kinematics in unilateral cerebral palsy demonstrates preserved independent neural control of each limb. *Front. Hum. Neurosci.* 11:50. 10.3389/fnhum.2017.00050 28243195 PMC5303755

[B24] Buller A. J. Eccles J. C. Eccles R. M. (1960). Differentiation of fast and slow muscles in the cat hind limb. *J. Physiol. (Lond.)* 150 399–416. 10.1113/jphysiol.1960.sp006394 13805873 PMC1363171

[B25] Campbell S. K. Gaebler-Spira D. Zawacki L. Clark A. Boynewicz K. deRegnier R.-A. (2012). Effects on motor development of kicking and stepping exercise in preterm infants with periventricular brain injury: a pilot study. *J. Pediatr. Rehabil. Med.* 5 15–27. 10.3233/prm-2011-0185 22543889 PMC3584696

[B26] Capaday C. (2002). The special nature of human walking and its neural control. *Trends Neurosci.* 25 370–376. 10.1016/s0166-2236(02)02173-2 12079766

[B27] Cappellini G. Ivanenko Y. P. Martino G. MacLellan M. J. Sacco A. Morelli D. (2016). Immature spinal locomotor output in children with cerebral palsy. *Front. Physiol.* 7:478. 10.3389/fphys.2016.00478 27826251 PMC5078720

[B28] Cappellini G. Sylos-Labini F. MacLellan M. Assenza C. Libernini L. Morelli D. (2020). Locomotor patterns during obstacle avoidance in children with Cerebral Palsy. *J. Neurophysiol.* 10.1152/jn.00163.2020 [Epub ahead of print]. 32667246

[B29] Cappellini G. Sylos-Labini F. MacLellan M. J. Sacco A. Morelli D. Lacquaniti F. (2018). Backward walking highlights gait asymmetries in children with cerebral palsy. *J. Neurophysiol.* 119 1153–1165. 10.1152/jn.00679.2017 29357466

[B30] Cavarsan C. F. Gorassini M. A. Quinlan K. A. (2019). Animal models of developmental motor disorders: parallels to human motor dysfunction in cerebral palsy. *J. Neurophysiol.* 122 1238–1253. 10.1152/jn.00233.2019 31411933 PMC6766736

[B31] Chakrabarty S. Shulman B. Martin J. H. (2009). Activity-dependent codevelopment of the corticospinal system and target interneurons in the cervical spinal cord. *J. Neurosci.* 29 8816–8827. 10.1523/jneurosci.0735-09.2009 19587289 PMC3849701

[B32] Christensen D. Van Naarden Braun K. Doernberg N. S. Maenner M. J. Arneson C. L. Durkin M. S. (2014). Prevalence of cerebral palsy, co-occurring autism spectrum disorders, and motor functioning – autism and developmental disabilities monitoring network, USA, 2008. *Dev. Med. Child Neurol.* 56 59–65. 10.1111/dmcn.12268 24117446 PMC4351771

[B33] Christiansen L. Lundbye-Jensen J. Perez M. A. Nielsen J. B. (2017). How plastic are human spinal cord motor circuitries? *Exp. Brain Res.* 235 3243–3249. 10.1007/s00221-017-5037-x 28776155

[B34] Clowry G. J. (2007). The dependence of spinal cord development on corticospinal input and its significance in understanding and treating spastic cerebral palsy. *Neurosci. Biobehav. Rev.* 31 1114–1124. 10.1016/j.neubiorev.2007.04.007 17544509

[B35] Condliffe E. G. Jeffery D. T. Emery D. J. Gorassini M. A. (2016). Spinal inhibition and motor function in adults with spastic cerebral palsy. *J. Physiol. (Lond.)* 594 2691–2705. 10.1113/jp271886 26842905 PMC4865571

[B36] Crenna P. (1998). Spasticity and “spastic” gait in children with cerebral palsy. *Neurosci. Biobehav. Rev.* 22 571–578. 10.1016/s0149-7634(97)00046-8 9595571

[B37] Currie S. P. Sillar K. T. (2018). Developmental changes in spinal neuronal properties, motor network configuration, and neuromodulation at free-swimming stages of Xenopus tadpoles. *J. Neurophysiol.* 119 786–795. 10.1152/jn.00219.2017 29142093 PMC5899306

[B38] Damiano D. (2015). Muscle synergies: input or output variables for neural control? *Dev. Med. Child Neurol.* 57 1091–1092. 10.1111/dmcn.12843 26195185 PMC4715471

[B39] Dan B. Bouillot E. Mewasingh L. D. Devalck C. Bengoetxea A. Christophe C. (2004). Gait control in spinal palsy. *Brain Dev.* 26 463–468. 15351083 10.1016/j.braindev.2004.01.003

[B40] Davis B. L. Vaughan C. L. (1993). Phasic behavior of EMG signals during gait: Use of multivariate statistics. *J. Electromyogr. Kinesiol.* 3 51–60. 10.1016/1050-6411(93)90023-p 20719624

[B41] Day J. A. Fox E. J. Lowe J. Swales H. B. Behrman A. L. (2004). Locomotor training with partial body weight support on a treadmill in a nonambulatory child with spastic tetraplegic cerebral palsy: a case report. *Pediatr. Phys. Ther.* 16 106–113. 10.1097/01.pep.0000127569.83372.c8 17057535

[B42] Dayanidhi S. Kutch J. J. Valero-Cuevas F. J. (2013). Decrease in muscle contraction time complements neural maturation in the development of dynamic manipulation. *J. Neurosci.* 33 15050–15055. 10.1523/jneurosci.1968-13.2013 24048835 PMC3776057

[B43] Degelean M. De Borre L. Salvia P. Pelc K. Kerckhofs E. De Meirleir L. (2012). Effect of ankle-foot orthoses on trunk sway and lower limb intersegmental coordination in children with bilateral cerebral palsy. *J. Pediatr. Rehabil. Med.* 5 171–179. 10.3233/prm-2012-0209 23023249

[B44] Dehorter N. Vinay L. Hammond C. Ben-Ari Y. (2012). Timing of developmental sequences in different brain structures: physiological and pathological implications. *Eur. J. Neurosci.* 35 1846–1856. 10.1111/j.1460-9568.2012.08152.x 22708595

[B45] Dekopov A. V. Shabalov V. A. Tomsky A. A. Hit M. V. Salova E. M. (2015). Chronic spinal cord stimulation in the treatment of cerebral and spinal spasticity. *Stereotact. Funct. Neurosurg.* 93 133–139. 25765082 10.1159/000368905

[B46] Delabastita T. Desloovere K. Meyns P. (2016). Restricted arm swing affects gait stability and increased walking speed alters trunk movements in children with cerebral palsy. *Front. Hum. Neurosci.* 10:354. 10.3389/fnhum.2016.00354 27471457 PMC4945643

[B47] Denny-Brown D. E. (1929). The histological features of striped muscle in relation to its functional activity. *Proc. R. Soc. Lond. Ser. B* 104 371–411. 10.1098/rspb.1929.0014

[B48] Deon L. L. Gaebler-Spira D. (2010). Assessment and treatment of movement disorders in children with cerebral palsy. *Orthop. Clin. North Am.* 41 507–517. 10.1016/j.ocl.2010.06.001 20868881

[B49] Dewolf A. H. Sylos-Labini F. Cappellini G. Lacquaniti F. Ivanenko Y. (2020). Emergence of different gaits in infancy: relationship between developing neural circuitries and changing biomechanics. *Front. Bioeng. Biotechnol.* 8:473. 10.3389/fbioe.2020.00473 32509753 PMC7248179

[B50] Dietz V. Müller R. (2004). Degradation of neuronal function following a spinal cord injury: mechanisms and countermeasures. *Brain* 127 2221–2231. 10.1093/brain/awh255 15269117

[B51] Dixon P. C. Stebbins J. Theologis T. Zavatsky A. B. (2016). The use of turning tasks in clinical gait analysis for children with cerebral palsy. *Clin. Biomech. (Bristol, Avon)* 32 286–294. 10.1016/j.clinbiomech.2015.10.010 26549659

[B52] Dominici N. Ivanenko Y. P. Cappellini G. d’Avella A. Mondì V. Cicchese M. (2011). Locomotor primitives in newborn babies and their development. *Science* 334 997–999. 10.1126/science.1210617 22096202

[B53] Dominici N. Ivanenko Y. P. Cappellini G. Zampagni M. L. Lacquaniti F. (2010). Kinematic strategies in newly walking toddlers stepping over different support surfaces. *J. Neurophysiol.* 103 1673–1684. 10.1152/jn.00945.2009 20089810

[B54] Drew T. Andujar J.-E. Lajoie K. Yakovenko S. (2008). Cortical mechanisms involved in visuomotor coordination during precision walking. *Brain Res. Rev.* 57 199–211. 10.1016/j.brainresrev.2007.07.017 17935789

[B55] Drew T. Marigold D. S. (2015). Taking the next step: cortical contributions to the control of locomotion. *Curr. Opin. Neurobiol.* 33C 25–33. 10.1016/j.conb.2015.01.011 25643847

[B56] Drobyshevsky A. Quinlan K. A. (2017). Spinal cord injury in hypertonic newborns after antenatal hypoxia-ischemia in a rabbit model of cerebral palsy. *Exp. Neurol.* 293 13–26. 10.1016/j.expneurol.2017.03.017 28347765 PMC5509441

[B57] Drużbicki M. Rusek W. Snela S. Dudek J. Szczepanik M. Zak E. (2013). Functional effects of robotic-assisted locomotor treadmill thearapy in children with cerebral palsy. *J. Rehabil. Med.* 45 358–363. 10.2340/16501977-1114 23450428

[B58] Elnahhas A. M. Elshennawy S. Aly M. G. (2019). Effects of backward gait training on balance, gross motor function, and gait in children with cerebral palsy: a systematic review. *Clin. Rehabil.* 33 3–12. 10.1177/0269215518790053 30043634

[B59] Eyre J. A. Taylor J. P. Villagra F. Smith M. Miller S. (2001). Evidence of activity-dependent withdrawal of corticospinal projections during human development. *Neurology* 57, 1543–1554. 10.1212/WNL.57.9.1543 11706088

[B60] Falisse A. Pitto L. Kainz H. Hoang H. Wesseling M. Van Rossom S. (2020). Physics-based simulations to predict the differential effects of motor control and musculoskeletal deficits on gait dysfunction in cerebral palsy: a retrospective case study. *Front. Hum. Neurosci.* 14:40. 10.3389/fnhum.2020.00040 32132911 PMC7040166

[B61] Fetcho J. R. McLean D. L. (2010). Some principles of organization of spinal neurons underlying locomotion in zebrafish and their implications. *Ann. N. Y. Acad. Sci.* 1198 94–104. 10.1111/j.1749-6632.2010.05539.x 20536924 PMC3579554

[B62] Forma V. Anderson D. I. Provasi J. Soyez E. Martial M. Huet V. (2019). What does prone skateboarding in the newborn tell us about the ontogeny of human locomotion? *Child. Dev.* 90 1286–1302. 31267516 10.1111/cdev.13251

[B63] Forssberg H. (1985). Ontogeny of human locomotor control. I. Infant stepping, supported locomotion and transition to independent locomotion. *Exp. Brain Res.* 57 480–493. 3979491 10.1007/BF00237835

[B64] Forssberg H. (1999). Neural control of human motor development. *Curr. Opin. Neurobiol.* 9 676–682. 10.1016/s0959-4388(99)00037-9 10607646

[B65] Friel K. M. Martin J. H. (2007). Bilateral activity-dependent interactions in the developing corticospinal system. *J. Neurosci.* 27 11083–11090. 10.1523/jneurosci.2814-07.2007 17928450 PMC2740658

[B66] Friel K. M. Williams P. T. J. A. Serradj N. Chakrabarty S. Martin J. H. (2014). Activity-based therapies for repair of the corticospinal system injured during development. *Front. Neurol.* 5:229. 10.3389/fneur.2014.00229 25505443 PMC4241838

[B67] Fukson O. I. Berkinblit M. B. Feldman A. G. (1980). The spinal frog takes into account the scheme of its body during the wiping reflex. *Science* 209 1261–1263. 10.1126/science.7403886 7403886

[B68] Garwicz M. Christensson M. Psouni E. (2009). A unifying model for timing of walking onset in humans and other mammals. *PNAS* 106 21889–21893. 10.1073/pnas.0905777106 20018704 PMC2799813

[B69] Gazula V.-R. Roberts M. Luzzio C. Jawad A. F. Kalb R. G. (2004). Effects of limb exercise after spinal cord injury on motor neuron dendrite structure. *J. Comp. Neurol.* 476 130–145. 10.1002/cne.20204 15248194

[B70] Gill M. L. Grahn P. J. Calvert J. S. Linde M. B. Lavrov I. A. Strommen J. A. (2018). Neuromodulation of lumbosacral spinal networks enables independent stepping after complete paraplegia. *Nat. Med.* 24 1677–1682. 10.1038/s41591-018-0175-7 30250140

[B71] Gormley M. E. (2001). Treatment of neuromuscular and musculoskeletal problems in cerebral palsy. *Pediatr. Rehabil.* 4 5–16. 10.1080/13638490151068393 11330850

[B72] Gould N. Moreland M. Alvarez R. Trevino S. Fenwick J. (1989). Development of the child’s arch. *Foot Ankle* 9 241–245. 2731836 10.1177/107110078900900506

[B73] Graham H. K. Rosenbaum P. Paneth N. Dan B. Lin J.-P. Damiano D. L. (2016). Cerebral palsy. *Nat. Rev. Dis. Primers* 2:15082. 10.1038/nrdp.2015.82 PMC9619297 27188686

[B74] Grillner S. El Manira A. (2020). Current principles of motor control, with special reference to vertebrate locomotion. *Physiol. Rev.* 100 271–320. 10.1152/physrev.00015.2019 31512990

[B75] Hadders-Algra M. (2004). General movements: a window for early identification of children at high risk for developmental disorders. *J. Pediatr.* 145 S12–S18. 15292882 10.1016/j.jpeds.2004.05.017

[B76] Hadders-Algra M. (2014). Early diagnosis and early intervention in cerebral palsy. *Front. Neurol.* 5:185. 10.3389/fneur.2014.00185 25309506 PMC4173665

[B77] Hadders-Algra M. (2018). Early human motor development: From variation to the ability to vary and adapt. *Neurosci. Biobehav. Rev.* 90 411–427. 10.1016/j.neubiorev.2018.05.009 29752957

[B78] Handsfield G. G. Meyer C. H. Abel M. F. Blemker S. S. (2016). Heterogeneity of muscle sizes in the lower limbs of children with cerebral palsy. *Muscle Nerve* 53 933–945. 10.1002/mus.24972 26565390

[B79] Hansen N. L. Conway B. A. Halliday D. M. Hansen S. Pyndt H. S. Biering-Sørensen F. (2005). Reduction of common synaptic drive to ankle dorsiflexor motoneurons during walking in patients with spinal cord lesion. *J. Neurophysiol.* 94 934–942. 10.1152/jn.00082.2005 15800077

[B80] Hanson C. J. Jones L. J. (1989). Gait abnormalities and inhibitive casts in cerebral palsy. Literature review. *J. Am. Podiatr. Med. Assoc.* 79 53–59. 10.7547/87507315-79-2-53 2659762

[B81] Hashiguchi Y. Ohata K. Osako S. Kitatani R. Aga Y. Masaki M. (2018). Number of synergies is dependent on spasticity and gait kinetics in children with cerebral palsy. *Pediatr. Phys. Ther.* 30 34–38. 10.1097/pep.0000000000000460 29252834

[B82] Heng C. de Leon R. D. (2007). The rodent lumbar spinal cord learns to correct errors in hindlimb coordination caused by viscous force perturbations during stepping. *J. Neurosci.* 27 8558–8562. 10.1523/jneurosci.1635-07.2007 17687033 PMC6672945

[B83] Hoogkamer W. Meyns P. Duysens J. (2014). Steps forward in understanding backward gait: from basic circuits to rehabilitation. *Exerc. Sport Sci. Rev.* 42 23–29. 10.1249/jes.0000000000000000 24188982

[B84] Hösl M. Böhm H. Eck J. Döderlein L. Arampatzis A. (2018). Effects of backward-downhill treadmill training versus manual static plantarflexor stretching on muscle-joint pathology and function in children with spastic Cerebral Palsy. *Gait Posture* 65 121–128. 10.1016/j.gaitpost.2018.07.171 30558918

[B85] Hug F. Turpin N. A. Dorel S. Guével A. (2012). Smoothing of electromyographic signals can influence the number of extracted muscle synergies. *Clin. Neurophysiol.* 123 1895–1896. 10.1016/j.clinph.2012.01.015 22342685

[B86] Hurd C. Livingstone D. Brunton K. Teves M. Zewdie E. Smith A. (2017). Early intensive leg training to enhance walking in children with perinatal stroke: protocol for a randomized controlled trial. *Phys. Ther.* 97 818–825. 10.1093/ptj/pzx045 28789469

[B87] Hutton J. L. Pharoah P. O. (2002). Effects of cognitive, motor, and sensory disabilities on survival in cerebral palsy. *Arch. Dis. Child* 86 84–89. 10.1136/adc.86.2.84 11827899 PMC1761069

[B88] Ivanenko Y. P. Cappellini G. Dominici N. Poppele R. E. Lacquaniti F. (2005). Coordination of locomotion with voluntary movements in humans. *J. Neurosci.* 25 7238–7253. 10.1523/jneurosci.1327-05.2005 16079406 PMC6725226

[B89] Ivanenko Y. P. Dominici N. Cappellini G. Dan B. Cheron G. Lacquaniti F. (2004). Development of pendulum mechanism and kinematic coordination from the first unsupported steps in toddlers. *J. Exp. Biol.* 207 3797–3810. 10.1242/jeb.01214 15371487

[B90] Ivanenko Y. P. Dominici N. Cappellini G. Di Paolo A Giannini C. Poppele R. E. (2013). Changes in the spinal segmental motor output for stepping during development from infant to adult. *J. Neurosci.* 33 3025a–3036a. 23407959 10.1523/JNEUROSCI.2722-12.2013 PMC6619203

[B91] Ivanenko Y. P. Dominici N. Daprati E. Nico D. Cappellini G. Lacquaniti F. (2011). Locomotor body scheme. *Hum. Mov. Sci.* 30 341–351. 10.1016/j.humov.2010.04.001 21453667

[B92] Ivanenko Y. P. Grasso R. Macellari V. Lacquaniti F. (2002). Control of foot trajectory in human locomotion: role of ground contact forces in simulated reduced gravity. *J. Neurophysiol.* 87 3070–3089. 10.1152/jn.2002.87.6.3070 12037209

[B93] Ivanenko Y. P. Gurfinkel V. S. Selionov V. A. Solopova I. A. Sylos-Labini F. Guertin P. A. (2017). Tonic and rhythmic spinal activity underlying locomotion. *Curr. Pharm. Des.* 23 1753–1763. 10.2174/1381612823666170125152246 28128063

[B94] Jankowska E. Padel Y. Tanaka R. (1976). Disynaptic inhibition of spinal motoneurones from the motor cortex in the monkey. *J. Physiol. (Lond.)* 258 467–487. 10.1113/jphysiol.1976.sp011431 822152 PMC1308987

[B95] Jiang Y.-Q. Sarkar A. Amer A. Martin J. H. (2018). Transneuronal downregulation of the premotor cholinergic system after corticospinal tract loss. *J. Neurosci.* 38 8329–8344. 10.1523/jneurosci.3410-17.2018 30049887 PMC6158693

[B96] Kaas J. H. (2005). From mice to men: the evolution of the large, complex human brain. *J. Biosci.* 30 155–165. 10.1007/bf02703695 15886451

[B97] Kiehn O. (2016). Decoding the organization of spinal circuits that control locomotion. *Nat. Rev. Neurosci.* 17 224–238. 10.1038/nrn.2016.9 26935168 PMC4844028

[B98] Kim W.-H. Kim W.-B. Yun C.-K. (2016). The effects of forward and backward walking according to treadmill inclination in children with cerebral palsy. *J. Phys. Ther. Sci.* 28 1569–1573. 10.1589/jpts.28.1569 27313373 PMC4905912

[B99] Kim Y. Bulea T. C. Damiano D. L. (2018). Children with cerebral palsy have greater stride-to-stride variability of muscle synergies during gait than typically developing children: implications for motor control complexity. *Neurorehabil. Neural Repair* 32 834–844. 10.1177/1545968318796333 30223739 PMC7271466

[B100] Kolobe T. H. A. Fagg A. H. (2019). Robot reinforcement and error-based movement learning in infants with and without cerebral palsy. *Phys. Ther.* 99 677–688. 10.1093/ptj/pzz043 31155667 PMC6545273

[B101] Koman L. A. Paterson Smith B. Balkrishnan R. (2003). Spasticity associated with cerebral palsy in children: guidelines for the use of botulinum A toxin. *Paediatr. Drugs* 5 11–23. 10.2165/00128072-200305010-00002 12513103

[B102] Kudo N. Furukawa F. Okado N. (1993). Development of descending fibers to the rat embryonic spinal cord. *Neurosci. Res.* 16 131–141. 10.1016/0168-0102(93)90080-a 8387169

[B103] Kuenzle C. Brunner R. (2009). The effects of the norsk funktion-walking orthosis on the walking ability of children with cerebral palsy and severe gait impairment. *J. Prosthetics Orthotics* 21 138–144. 10.1097/jpo.0b013e3181b173ec

[B104] La Scaleia V. Ivanenko Y. Fabiano A. Sylos-Labini F. Cappellini G. Picone S. (2018). Early manifestation of arm-leg coordination during stepping on a surface in human neonates. *Exp. Brain Res.* 236 1105–1115. 10.1007/s00221-018-5201-y 29441470

[B105] Lacquaniti F. Ivanenko Y. P. Zago M. (2012a). Development of human locomotion. *Curr. Opin. Neurobiol.* 22 822–828. 22498713 10.1016/j.conb.2012.03.012

[B106] Lacquaniti F. Ivanenko Y. P. Zago M. (2012b). Patterned control of human locomotion. *J. Physiol. (Lond.)* 590 2189–2199. 10.1113/jphysiol.2011.215137 22411012 PMC3424743

[B107] Lajoie K. Andujar J.-E. Pearson K. Drew T. (2010). Neurons in area 5 of the posterior parietal cortex in the cat contribute to interlimb coordination during visually guided locomotion: a role in working memory. *J. Neurophysiol.* 103 2234–2254. 10.1152/jn.01100.2009 20386041

[B108] Law L. S. H. Webb C. Y. (2005). Gait adaptation of children with cerebral palsy compared with control children when stepping over an obstacle. *Dev. Med. Child Neurol.* 47 321–328. 10.1017/s0012162205000617 15892374

[B109] Leigh S. R. (2004). Brain growth, life history, and cognition in primate and human evolution. *Am. J. Primatol.* 62 139–164. 10.1002/ajp.20012 15027089

[B110] Leonard C. T. Hirschfeld H. Forssberg H. (1991). The development of independent walking in children with cerebral palsy. *Dev. Med. Child Neurol.* 33 567–577. 10.1111/j.1469-8749.1991.tb14926.x 1879620

[B111] Lerner Z. F. Damiano D. L. Bulea T. C. (2017). The effects of exoskeleton assisted knee extension on lower-extremity gait kinematics, kinetics, and muscle activity in children with cerebral palsy. *Sci. Rep.* 7:13512. 10.1038/s41598-017-13554-2 PMC5647342 29044202

[B112] Levchenkova V. D. Semenova K. A. (2012). Contemporary views of the morphological basis of infant cerebral palsy. *Zh Nevrol Psikhiatr Im S S Korsakova* 112 4–8. 23330184

[B113] Levine A. J. Hinckley C. A. Hilde K. L. Driscoll S. P. Poon T. H. Montgomery J. M. (2014). Identification of a cellular node for motor control pathways. *Nat. Neurosci.* 17 586–593. 10.1038/nn.3675 24609464 PMC4569558

[B114] Lewerenz A. Wolf S. I. Dreher T. Krautwurst B. K. (2019). Performance of stair negotiation in patients with cerebral palsy and stiff knee gait. *Gait Posture* 71 14–19. 10.1016/j.gaitpost.2019.04.005 30999269

[B115] Li T. Chen X. Cao S. Zhang X. Chen X. (2019). Human hands-and-knees crawling movement analysis based on time-varying synergy and synchronous synergy theories. *Math. Biosci. Eng.* 16 2492–2513. 10.3934/mbe.2019125 31137224

[B116] Lieber R. L. Fridén J. (2019). Muscle contracture and passive mechanics in cerebral palsy. *J. Appl. Physiol.* 126 1492–1501. 10.1152/japplphysiol.00278.2018 30571285 PMC6589815

[B117] Lorentzen J. Willerslev-Olsen M. Hüche Larsen H. Farmer S. F. Nielsen J. B. (2019). Maturation of feedforward toe walking motor program is impaired in children with cerebral palsy. *Brain* 142 526–541. 10.1093/brain/awz002 30726881

[B118] Maier E. (1961). Longitudinal measurement research on the maturation of the child’s foot. *Monatsschr Kinderheilkd* 109 222–226. 13765434

[B119] Malone A. Kiernan D. French H. Saunders V. O’Brien T. (2016). Obstacle crossing during gait in children with cerebral palsy: cross-sectional study with kinematic analysis of dynamic balance and trunk control. *Phys. Ther.* 96 1208–1215. 10.2522/ptj.20150360 26893506

[B120] Martin J. H. (2005). The corticospinal system: from development to motor control. *Neuroscientist* 11 161–173. 10.1177/1073858404270843 15746384

[B121] Martino G. Ivanenko Y. P. d’Avella A. Serrao M. Ranavolo A. Draicchio F. (2015). Neuromuscular adjustments of gait associated with unstable conditions. *J. Neurophysiol.* 114 2867–2882. 10.1152/jn.00029.2015 26378199 PMC4737426

[B122] Massaad F. Lejeune T. M. Detrembleur C. (2010). Reducing the energy cost of hemiparetic gait using center of mass feedback: a pilot study. *Neurorehabil. Neural Repair* 24 338–347. 10.1177/1545968309349927 19890020

[B123] Mathewson M. A. Lieber R. L. (2015). Pathophysiology of muscle contractures in cerebral palsy. *Phys. Med. Rehabil. Clin. N Am.* 26 57–67. 10.1016/j.pmr.2014.09.005 25479779 PMC4258234

[B124] Mawase F. Bar-Haim S. Joubran K. Rubin L. Karniel A. Shmuelof L. (2016). Increased adaptation rates and reduction in trial-by-trial variability in subjects with cerebral palsy following a multi-session locomotor adaptation training. *Front. Hum. Neurosci.* 10:203. 10.3389/fnhum.2016.00203 27199721 PMC4854882

[B125] McNevin N. H. Coraci L. Schafer J. (2000). Gait in adolescent cerebral palsy: the effect of partial unweighting. *Arch. Phys. Med. Rehabil.* 81 525–528. 10.1053/mr.2000.4429 10768548

[B126] Meyns P. Desloovere K. Van Gestel L. Massaad F. Smits-Engelsman B. Duysens J. (2012). Altered arm posture in children with cerebral palsy is related to instability during walking. *Eur. J. Paediatr. Neurol.* 16 528–535. 10.1016/j.ejpn.2012.01.011 22336190

[B127] Meyns P. Duysens J. Desloovere K. (2016). The arm posture in children with unilateral Cerebral Palsy is mainly related to antero-posterior gait instability. *Gait Posture* 49 132–135. 10.1016/j.gaitpost.2016.06.033 27414040

[B128] Meyns P. Molenaers G. Duysens J. Jonkers I. (2017). The differential effect of arm movements during gait on the forward acceleration of the centre of mass in children with cerebral palsy and typically developing children. *Front. Hum. Neurosci.* 11:96. 10.3389/fnhum.2017.00096 28298890 PMC5331063

[B129] Mima T. Hallett M. (1999). Corticomuscular coherence: a review. *J. Clin. Neurophysiol.* 16 501–511. 10600018 10.1097/00004691-199911000-00002

[B130] Minassian K. Hofstoetter U. S. Dzeladini F. Guertin P. A. Ijspeert A. (2017). The human central pattern generator for locomotion. *Neuroscientist* 23 649–663. 28351197 10.1177/1073858417699790

[B131] Moreau N. G. Simpson K. N. Teefey S. A. Damiano D. L. (2010). Muscle architecture predicts maximum strength and is related to activity levels in cerebral palsy. *Phys. Ther.* 90 1619–1630. 10.2522/ptj.20090377 20847035 PMC2967708

[B132] Morrell D. S. Pearson J. M. Sauser D. D. (2002). Progressive bone and joint abnormalities of the spine and lower extremities in cerebral palsy. *Radiographics* 22 257–268. 10.1148/radiographics.22.2.g02mr19257 11896216

[B133] Myklebust B. M. (1990). A review of myotatic reflexes and the development of motor control and gait in infants and children: a special communication. *Phys. Ther.* 70 188–203. 10.1093/ptj/70.3.188 2304976

[B134] Myklebust B. M. Gottlieb G. L. Penn R. D. Agarwal G. C. (1982). Reciprocal excitation of antagonistic muscles as a differentiating feature in spasticity. *Ann. Neurol.* 12 367–374. 10.1002/ana.410120409 7149662

[B135] Nemanich S. T. Mueller B. A. Gillick B. T. (2019). Neurite orientation dispersion and density imaging quantifies corticospinal tract microstructural organization in children with unilateral cerebral palsy. *Hum. Brain Mapp.* 40 4888–4900. 10.1002/hbm.24744 31355991 PMC6813864

[B136] Nielsen J. B. Brittain J.-S. Halliday D. M. Marchand-Pauvert V. Mazevet D. Conway B. A. (2008). Reduction of common motoneuronal drive on the affected side during walking in hemiplegic stroke patients. *Clin. Neurophysiol.* 119 2813–2818. 10.1016/j.clinph.2008.07.283 18848803

[B137] Noble J. J. (2014). *Musculoskeletal and Spinal Cord Imaging in Bilateral Spastic Cerebral Palsy.* Doctoral dissertation, King’s College London, London.

[B138] Noble J. J. Fry N. Lewis A. P. Charles-Edwards G. D. Keevil S. F. Gough M. (2014). Bone strength is related to muscle volume in ambulant individuals with bilateral spastic cerebral palsy. *Bone* 66 251–255. 10.1016/j.bone.2014.06.028 24984277

[B139] Novak I. Morgan C. Adde L. Blackman J. Boyd R. N. Brunstrom-Hernandez J. (2017). Early, accurate diagnosis and early intervention in cerebral palsy: advances in diagnosis and treatment. *JAMA Pediatr.* 171 897–907. 28715518 10.1001/jamapediatrics.2017.1689 PMC9641643

[B140] Oberhofer K. Stott N. S. Mithraratne K. Anderson I. A. (2010). Subject-specific modelling of lower limb muscles in children with cerebral palsy. *Clin. Biomech. (Bristol, Avon)* 25 88–94. 10.1016/j.clinbiomech.2009.09.007 19836868

[B141] Oliveira L. C. Trócoli T. O. Kanashiro M. S. Braga D. Cyrillo F. N. (2014). Electromyographic analysis of rectus femoris activity during seated to standing position and walking in water and on dry land in healthy children and children with cerebral palsy. *J. Electromyogr. Kinesiol.* 24 855–859. 10.1016/j.jelekin.2014.08.008 25282573

[B142] Papadelis C. Kaye H. Shore B. Snyder B. Grant P. E. Rotenberg A. (2019). Maturation of corticospinal tracts in children with hemiplegic cerebral palsy assessed by diffusion tensor imaging and transcranial magnetic stimulation. *Front. Hum. Neurosci.* 13:254. 10.3389/fnhum.2019.00254 31396066 PMC6668599

[B143] Patterson K. K. Gage W. H. Brooks D. Black S. E. McIlroy W. E. (2010). Evaluation of gait symmetry after stroke: a comparison of current methods and recommendations for standardization. *Gait Posture* 31 241–246. 10.1016/j.gaitpost.2009.10.014 19932621

[B144] Pearson K. Gramlich R. (2010). Updating neural representations of objects during walking. *Ann. N. Y. Acad. Sci.* 1198 1–9. 10.1111/j.1749-6632.2009.05422.x 20536915

[B145] Perreault M.-C. Glover J. C. (2013). Glutamatergic reticulospinal neurons in the mouse: developmental origins, axon projections, and functional connectivity. *Ann. N. Y. Acad. Sci.* 1279 80–89. 10.1111/nyas.12054 23531005

[B146] Petersen T. H. Farmer S. F. Kliim-Due M. Nielsen J. B. (2013). Failure of normal development of central drive to ankle dorsiflexors relates to gait deficits in children with cerebral palsy. *J. Neurophysiol.* 109 625–639. 10.1152/jn.00218.2012 23136346

[B147] Pitto L. van Rossom S. Desloovere K. Molenaers G. Huenaerts C. De Groote F. (2020). Pre-treatment EMG can be used to model post-treatment muscle coordination during walking in children with cerebral palsy. *PLoS One* 15:e0228851. 10.1371/journal.pone.0228851 32050002 PMC7015404

[B148] Poon D. M. Y. Hui-Chan C. W. Y. (2009). Hyperactive stretch reflexes, co-contraction, and muscle weakness in children with cerebral palsy. *Dev. Med. Child Neurol.* 51 128–135. 10.1111/j.1469-8749.2008.03122.x 19018843

[B149] Poppele R. Bosco G. (2003). Sophisticated spinal contributions to motor control. *Trends Neurosci.* 26 269–276. 10.1016/s0166-2236(03)00073-0 12744844

[B150] Prosser L. A. Ohlrich L. B. Curatalo L. A. Alter K. E. Damiano D. L. (2012). Feasibility and preliminary effectiveness of a novel mobility training intervention in infants and toddlers with cerebral palsy. *Dev. Neurorehabil.* 15 259–266. 10.3109/17518423.2012.687782 22670679 PMC3594802

[B151] Rauscent A. Le Ray D. Cabirol-Pol M.-J. Sillar K. T. Simmers J. Combes D. (2006). Development and neuromodulation of spinal locomotor networks in the metamorphosing frog. *J. Physiol. Paris* 100 317–327. 10.1016/j.jphysparis.2007.05.009 17629683

[B152] Redd C. B. Barber L. A. Boyd R. N. Varnfield M. Karunanithi M. K. (2019). Development of a wearable sensor network for quantification of infant general movements for the diagnosis of cerebral palsy. *Conf. Proc. IEEE Eng. Med. Biol. Soc.* 2019 7134–7139. 31947480 10.1109/EMBC.2019.8857377

[B153] Reid L. B. Rose S. E. Boyd R. N. (2015). Rehabilitation and neuroplasticity in children with unilateral cerebral palsy. *Nat. Rev. Neurol.* 11 390–400. 10.1038/nrneurol.2015.97 26077839

[B154] Rethlefsen S. A. Blumstein G. Kay R. M. Dorey F. Wren T. A. L. (2017). Prevalence of specific gait abnormalities in children with cerebral palsy revisited: influence of age, prior surgery, and gross motor function classification system level. *Dev. Med. Child Neurol.* 59 79–88. 10.1111/dmcn.13205 27421715

[B155] Richards C. L. Malouin F. Dumas F. Marcoux S. Lepage C. Menier C. (1997). Early and intensive treadmill locomotor training for young children with cerebral palsy: a feasibility study. *Pediatr. Phys. Ther.* 9 158–165.

[B156] Ritterband-Rosenbaum A. Herskind A. Li X. Willerslev-Olsen M. Olsen M. D. Farmer S. F. (2017). A critical period of corticomuscular and EMG–EMG coherence detection in healthy infants aged 9–25 weeks. *J. Physiol.* 595 2699–2713. 10.1113/jp273090 28004392 PMC5390881

[B157] Rosenbaum P. Eliasson A.-C. Hidecker M. J. C. Palisano R. J. (2014). Classification in childhood disability: focusing on function in the 21st century. *J. Child Neurol.* 29 1036–1045. 10.1177/0883073814533008 24810083

[B158] Russo M. D’Andola M. Portone A. Lacquaniti F. d’Avella A. (2014). Dimensionality of joint torques and muscle patterns for reaching. *Front. Comput. Neurosci.* 8:24. 10.3389/fncom.2014.00024 24624078 PMC3939605

[B159] Salenius S. Salmelin R. Neuper C. Pfurtscheller G. Hari R. (1996). Human cortical 40 Hz rhythm is closely related to EMG rhythmicity. *Neurosci. Lett.* 213 75–78. 10.1016/0304-3940(96)12796-8 8858612

[B160] Sanger T. D. (2003). Pathophysiology of pediatric movement disorders. *J. Child Neurol.* 18(Suppl 1) S9–S24. 13677568 10.1177/0883073803018001S0401

[B161] Shabalov V. A. Dekopov A. V. Troshina E. M. (2006). Preliminary results of treatment for spastic forms of infantile cerebral paralysis by chronic epidural neurostimulation of lumbar enlargement. *Zh Vopr Neirokhir Im N N Burdenko* 3 10–13; discussion 13. 17125072

[B162] Short M. R. Damiano D. L. Kim Y. Bulea T. C. (2020). Children with unilateral cerebral palsy utilize more cortical resources for similar motor output during treadmill gait. *Front. Hum. Neurosci.* 14:36. 10.3389/fnhum.2020.00036 32153376 PMC7047842

[B163] Shuman B. Goudriaan M. Bar-On L. Schwartz M. H. Desloovere K. Steele K. M. (2016). Repeatability of muscle synergies within and between days for typically developing children and children with cerebral palsy. *Gait Posture* 45 127–132. 10.1016/j.gaitpost.2016.01.011 26979894

[B164] Shuman B. R. Goudriaan M. Desloovere K. Schwartz M. H. Steele K. M. (2018). Associations between muscle synergies and treatment outcomes in cerebral palsy are robust across clinical centers. *Arch. Phys. Med. Rehabil.* 99 2175–2182. 10.1016/j.apmr.2018.03.006 29649451 PMC6179956

[B165] Shuman B. R. Goudriaan M. Desloovere K. Schwartz M. H. Steele K. M. (2019a). Muscle synergies demonstrate only minimal changes after treatment in cerebral palsy. *J. Neuroeng. Rehabil.* 16:46. 10.1186/s12984-019-0502-3 PMC6441188 30925882

[B166] Shuman B. R. Goudriaan M. Desloovere K. Schwartz M. H. Steele K. M. (2019b). Muscle synergy constraints do not improve estimates of muscle activity from static optimization during gait for unimpaired children or children with cerebral palsy. *Front. Neurorobot.* 13:102. 10.3389/fnbot.2019.00102 31920612 PMC6927914

[B167] Shuman B. R. Schwartz M. H. Steele K. M. (2017). Electromyography data processing impacts muscle synergies during gait for unimpaired children and children with cerebral palsy. *Front. Comput. Neurosci.* 11:50. 10.3389/fncom.2017.00050 28634449 PMC5460588

[B168] Sidiropoulos A. N. Chen S. Kaminski T. R. M. Gordon A. M. (2019). Modulation of gait inter-limb coordination in children with unilateral spastic cerebral palsy after intensive upper extremity intervention. *Exp. Brain Res.* 237 1409–1419. 10.1007/s00221-019-05501-6 30888460

[B169] Smania N. Bonetti P. Gandolfi M. Cosentino A. Waldner A. Hesse S. (2011). Improved gait after repetitive locomotor training in children with cerebral palsy. *Am. J. Phys. Med. Rehabil.* 90 137–149. 10.1097/phm.0b013e318201741e 21217461

[B170] Smania N. Gandolfi M. Marconi V. Calanca A. Geroin C. Piazza S. (2012). Applicability of a new robotic walking aid in a patient with cerebral palsy. Case report. *Eur. J. Phys. Rehabil. Med.* 48 147–153. 22543558

[B171] Smith C. C. Paton J. F. R. Chakrabarty S. Ichiyama R. M. (2017). Descending systems direct development of key spinal motor circuits. *J. Neurosci.* 37 6372–6387. 10.1523/jneurosci.0149-17.2017 28576940 PMC6705699

[B172] Solopova I. A. Selionov V. A. Zhvansky D. S. Gurfinkel V. S. Ivanenko Y. (2016). Human cervical spinal cord circuitry activated by tonic input can generate rhythmic arm movements. *J. Neurophysiol.* 115 1018–1030. 10.1152/jn.00897.2015 26683072

[B173] Solopova I. A. Sukhotina I. A. Zhvansky D. S. Ikoeva G. A. Vissarionov S. V. Baindurashvili A. G. (2017). Effects of spinal cord stimulation on motor functions in children with cerebral palsy. *Neurosci. Lett.* 639 192–198. 10.1016/j.neulet.2017.01.003 28063935

[B174] Solopova I. A. Zhvansky D. S. Dolinskaya I. Y. Keshishian E. S. Selionov V. A. Sylos-Labini F. (2019). Muscle responses to passive joint movements in infants during the first year of life. *Front. Physiol.* 10:1158. 10.3389/fphys.2019.01158 31607940 PMC6769424

[B175] Steele K. M. Munger M. E. Peters K. M. Shuman B. R. Schwartz M. H. (2019). Repeatability of electromyography recordings and muscle synergies during gait among children with cerebral palsy. *Gait Posture* 67 290–295. 10.1016/j.gaitpost.2018.10.009 30396059 PMC6283402

[B176] Steele K. M. Rozumalski A. Schwartz M. H. (2015). Muscle synergies and complexity of neuromuscular control during gait in cerebral palsy. *Dev. Med. Child Neurol.* 57 1176–1182. 10.1111/dmcn.12826 26084733 PMC4683117

[B177] Steele K. M. Tresch M. C. Perreault E. J. (2013). The number and choice of muscles impact the results of muscle synergy analyses. *Front. Comput. Neurosci.* 7:105. 10.3389/fncom.2013.00105 23964232 PMC3737463

[B178] Sundström E. Kölare S. Souverbie F. Samuelsson E. B. Pschera H. Lunell N. O. (1993). Neurochemical differentiation of human bulbospinal monoaminergic neurons during the first trimester. *Brain Res. Dev. Brain Res.* 75 1–12. 10.1016/0165-3806(93)90059-j 7900931

[B179] Sutherland D. H. Davids J. R. (1993). Common gait abnormalities of the knee in cerebral palsy. *Clin. Orthop. Relat. Res.* 288 139–147. 8458127

[B180] Sylos-Labini F. Ivanenko Y. P. Maclellan M. J. Cappellini G. Poppele R. E. Lacquaniti F. (2014). Locomotor-like leg movements evoked by rhythmic arm movements in humans. *PLoS One* 9:e90775. 10.1371/journal.pone.0090775 24608249 PMC3946538

[B181] Sylos-Labini F. La Scaleia V. Cappellini G. Fabiano A. Picone S. Keshishian E. S. (2020). Distinct locomotor precursors in newborn babies. *Proc. Natl. Acad. Sci. U.S.A.* 117 9604–9612. 10.1073/pnas.1920984117 32284405 PMC7196819

[B182] Tang L. Li F. Cao S. Zhang X. Wu D. Chen X. (2015). Muscle synergy analysis in children with cerebral palsy. *J. Neural Eng.* 12:046017 10.1088/1741-2560/12/4/046017 26061115

[B183] Thelen E. (1995). Motor development: a new synthesis. *Am. Psychol.* 50 79–95. 10.1037/0003-066x.50.2.79 7879990

[B184] Thelen E. Cooke D. W. (1987). Relationship between newborn stepping and later walking: a new interpretation. *Dev. Med. Child Neurol.* 29 380–393. 10.1111/j.1469-8749.1987.tb02492.x 3596074

[B185] Valentín-Gudiol M. Mattern-Baxter K. Girabent-Farrés M. Bagur-Calafat C. Hadders-Algra M. Angulo-Barroso R. M. (2017). Treadmill interventions in children under six years of age at risk of neuromotor delay. *Cochrane Database Syst. Rev.* 7:CD009242. 10.1002/14651858.CD009242.pub3 PMC6483121 28755534

[B186] van den Brand R. Mignardot J.-B. von Zitzewitz J. Le Goff C. Fumeaux N. Wagner F. (2015). Neuroprosthetic technologies to augment the impact of neurorehabilitation after spinal cord injury. *Ann. Phys. Rehabil. Med.* 58 232–237. 10.1016/j.rehab.2015.04.003 26100230

[B187] Vinay L. Brocard F. Clarac F. Norreel J. C. Pearlstein E. Pflieger J. F. (2002). Development of posture and locomotion: an interplay of endogenously generated activities and neurotrophic actions by descending pathways. *Brain Res. Brain Res. Rev.* 40 118–129. 10.1016/s0165-0173(02)00195-9 12589911

[B188] von Zitzewitz J. Asboth L. Fumeaux N. Hasse A. Baud L. Vallery H. (2016). A neurorobotic platform for locomotor prosthetic development in rats and mice. *J. Neural Eng.* 13:026007 10.1088/1741-2560/13/2/026007 26860920

[B189] Wenger N. Moraud E. M. Gandar J. Musienko P. Capogrosso M. Baud L. (2016). Spatiotemporal neuromodulation therapies engaging muscle synergies improve motor control after spinal cord injury. *Nat. Med.* 22 138–145. 10.1038/nm.4025 26779815 PMC5061079

[B190] Willerslev-Olsen M. Lorentzen J. Sinkjaer T. Nielsen J. B. (2013). Passive muscle properties are altered in children with cerebral palsy before the age of 3 years and are difficult to distinguish clinically from spasticity. *Dev. Med. Child Neurol.* 55 617–623. 10.1111/dmcn.12124 23517272

[B191] Willerslev-Olsen M. Petersen T. H. Farmer S. F. Nielsen J. B. (2015). Gait training facilitates central drive to ankle dorsiflexors in children with cerebral palsy. *Brain* 138 589–603. 10.1093/brain/awu399 25623137 PMC4408439

[B192] Williams P. T. J. A. Jiang Y.-Q. Martin J. H. (2017). Motor system plasticity after unilateral injury in the developing brain. *Dev. Med. Child Neurol.* 59 1224–1229. 10.1111/dmcn.13581 28972274 PMC5773112

[B193] Williams S. E. Gibbs S. Meadows C. B. Abboud R. J. (2011). Classification of the reduced vertical component of the ground reaction force in late stance in cerebral palsy gait. *Gait Posture* 34 370–373. 10.1016/j.gaitpost.2011.06.003 21723132

[B194] Willoughby K. L. Dodd K. J. Shields N. (2009). A systematic review of the effectiveness of treadmill training for children with cerebral palsy. *Disabil. Rehabil.* 31 1971–1979. 10.3109/09638280902874204 19874075

[B195] Windhorst U. (1996b). On the role of recurrent inhibitory feedback in motor control. *Prog. Neurobiol.* 49 517–587. 10.1016/0301-0082(96)00023-8 8912393

[B196] Windhorst U. (1996a). The spinal cord and its brain: representations and models. To what extent do forebrain mechanisms appear at brainstem spinal cord levels? *Prog. Neurobiol.* 49 381–414. 10.1016/0301-0082(96)00022-6 8895994

[B197] Wingstrand M. Hägglund G. Rodby-Bousquet E. (2014). Ankle-foot orthoses in children with cerebral palsy: a cross sectional population based study of 2200 children. *BMC Musculoskelet. Disord.* 15:327. 10.1186/1471-2474-15-327 25274143 PMC4192348

[B198] Winter D. A. (1992). Foot trajectory in human gait: a precise and multifactorial motor control task. *Phys. Ther.* 72 45–53; discussion 54–56. 1728048 10.1093/ptj/72.1.45

[B199] Wonsetler E. C. Bowden M. G. (2017). A systematic review of mechanisms of gait speed change post-stroke. Part 1: spatiotemporal parameters and asymmetry ratios. *Top. Stroke Rehabil.* 24 435–446. 10.1080/10749357.2017.1285746 28220715 PMC5875693

[B200] Xiong Q. L. Wu X. Y. Yao J. Sukal T. M. Xiao N. Chen L. (2018). Inter-limb muscle synergy of hands-and-knees crawling in typical developing infants and infants with developmental delay. *Conf. Proc. IEEE Eng. Med. Biol. Soc.* 2018 4697–4700. 30441398 10.1109/EMBC.2018.8513123

[B201] Yakovenko S. Mushahwar V. VanderHorst V. Holstege G. Prochazka A. (2002). Spatiotemporal activation of lumbosacral motoneurons in the locomotor step cycle. *J. Neurophysiol.* 87 1542–1553. 10.1152/jn.00479.2001 11877525

[B202] Yakovlev P. Lecours A. (1967). “The myelogenetic cycles of regional maturation of the brain,” in *Regional Development of the Brain in Early Life*, ed. Minkowsky A. (Hoboken, NJ: Blackwell Scientific Publications), 3–70.

[B203] Yang J. F. Gorassini M. (2006). Spinal and brain control of human walking: implications for retraining of walking. *Neuroscientist* 12 379–389. 10.1177/1073858406292151 16957000

[B204] Yang J. F. Livingstone D. Brunton K. Kim D. Lopetinsky B. Roy F. (2013). Training to enhance walking in children with cerebral palsy: are we missing the window of opportunity? *Semin. Pediatr. Neurol.* 20 106–115. 10.1016/j.spen.2013.06.011 23948685

[B205] Yang J. F. Mitton M. Musselman K. E. Patrick S. K. Tajino J. (2015). Characteristics of the developing human locomotor system: similarities to other mammals. *Dev. Psychobiol.* 57 397–408. 10.1002/dev.21289 25754858

[B206] Yeo S. S. Jang S. H. Son S. M. (2014). The different maturation of the corticospinal tract and corticoreticular pathway in normal brain development: diffusion tensor imaging study. *Front. Hum. Neurosci.* 8:573. 10.3389/fnhum.2014.00573 25309378 PMC4163649

[B207] Yu Y. Chen X. Cao S. Wu D. Zhang X. Chen X. (2019). Gait synergetic neuromuscular control in children with cerebral palsy at different gross motor function classification system levels. *J. Neurophysiol.* 121 1680–1691. 10.1152/jn.00580.2018 30892974

[B208] Zehr E. P. Barss T. S. Dragert K. Frigon A. Vasudevan E. V. Haridas C. (2016). Neuromechanical interactions between the limbs during human locomotion: an evolutionary perspective with translation to rehabilitation. *Exp. Brain Res.* 234 3059–3081. 10.1007/s00221-016-4715-4 27421291 PMC5071371

[B209] Zehr E. P. Duysens J. (2004). Regulation of arm and leg movement during human locomotion. *Neuroscientist* 10 347–361. 10.1177/1073858404264680 15271262

[B210] Zelik K. E. La Scaleia V. Ivanenko Y. P. Lacquaniti F. (2014). Can modular strategies simplify neural control of multidirectional human locomotion? *J. Neurophysiol.* 111 1686–1702. 10.1152/jn.00776.2013 24431402

[B211] Zhu Z. Liu T. Li G. Li T. Inoue Y. (2015). Wearable sensor systems for infants. *Sensors (Basel)* 15 3721–3749. 10.3390/s150203721 25664432 PMC4367382

[B212] Zollinger M. Degache F. Currat G. Pochon L. Peyrot N. Newman C. J. (2016). External mechanical work and pendular energy transduction of overground and treadmill walking in adolescents with unilateral cerebral palsy. *Front. Physiol.* 7:121. 10.3389/fphys.2016.00121 27148062 PMC4829600

